# Oxytocin-Selective Nanogel Antibody Mimics

**DOI:** 10.3390/ijms23052534

**Published:** 2022-02-25

**Authors:** Rashmi Mahajan, Subramanian Suriyanarayanan, Gustaf D. Olsson, Jesper G. Wiklander, Teodor Aastrup, Börje Sellergren, Ian A. Nicholls

**Affiliations:** 1 Bioorganic and Biophysical Chemistry Laboratory, Linnaeus University Centre for Biomaterials Chemistry, Department of Chemistry and Biomedical Sciences, Linnaeus University, 39182 Kalmar, Sweden; rashmi.mahajan@lnu.se (R.M.); gustaf.olsson@lnu.se (G.D.O.); jesper.wiklander@lnu.se (J.G.W.); 2 Attana AB, Greta Arwidssons Väg 21, 11419 Stockholm, Sweden; teodor.aastrup@attana.com; 3 Biofilms Research Center for Biointerfaces, Department of Biomedical Sciences, Faculty of Health and Society, Malmö University, 20506 Malmö, Sweden; borje.sellergren@mau.se

**Keywords:** molecular dynamics, molecularly imprinted polymer, nanoparticle, NMR, peptide imprinting, plastic antibody, oxytocin, solid-phase synthesis, QCM

## Abstract

Oxytocin imprinted polymer nanoparticles were synthesized by glass bead supported solid phase synthesis, with NMR and molecular dynamics studies used to investigate monomer–template interactions. The nanoparticles were characterized by dynamic light scattering, scanning- and transmission electron microscopy and X-ray photoelectron spectroscopy. Investigation of nanoparticle-template recognition using quartz crystal microbalance-based studies revealed sub-nanomolar affinity, *k_d_* ≈ 0.3 ± 0.02 nM (standard error of the mean), comparable to that of commercial polyclonal antibodies, *k_d_* ≈ 0.02–0.2 nM.

## 1. Introduction

The selective detection of proteins and peptides is critical for the function of many clinical diagnostics and therapeutics, and for numerous processes in the pharmaceutical and biotechnology industries [1,2]. Antibodies are often used for protein detection due to the high specificities and selectivities they can display [3]; however, their production can be time-consuming and resource demanding [4]. Furthermore, their limited chemical and physical stabilities often lead to short shelf-lives and the need for refrigerated transport and storage [5]. Accordingly, alternative strategies for developing antibodies and materials with antibody-like properties have been pursued, including phage display [6,7], aptamer [8,9], and molecularly imprinted polymer (MIP) technologies [10,11,12].

The initial demonstration of antibody-like ligand specificities and selectivities in small molecule imprinted polymers [13] and the physical and chemical stabilities of these materials [14] has helped drive the search for new polymer compositions, design strategies, formats and synthesis methods suitable for use in the molecular imprinting of biomacromolecules [15,16]. The first reports of peptide molecularly imprinted nanoparticle synthesis using precipitation polymerization using water compatible polymer systems with low degrees of cross-linking [17,18] were a key development; however, nanoparticle yields and the general recognition site heterogeneity associated with molecularly imprinted materials remained issues. Moreover, entrapment is a problem with larger templates [19]. The subsequent development of molecularly imprinted polymer synthesis using biomacromolecular templates immobilized on the surfaces of flat substrates [19] and particles, e.g., beads [20] and nanoparticles [21], opened for the reuse of template materials [22]. The latter approach constituted a step change in the production of these materials as synthesis could be performed in a flow system, which allowed for up-scaling. Moreover, this type of system offers two distinct benefits; firstly, the release of the nanoparticles from the immobilized template constitutes an affinity purification step and, secondly, the potential for orientational control of template immobilization offers a means to reduce recognition site heterogeneity [23]. Imprinted nanoparticles obtained using this method can have dimensions comparable to those of proteins and can show fast binding kinetics and, in some cases, sub-nanomolar affinities for protein templates [24]. Collectively, these features make biomacromolecule imprinted nanoparticles of interest as substitutes for antibodies in a range of application areas.

**Figure 1:**
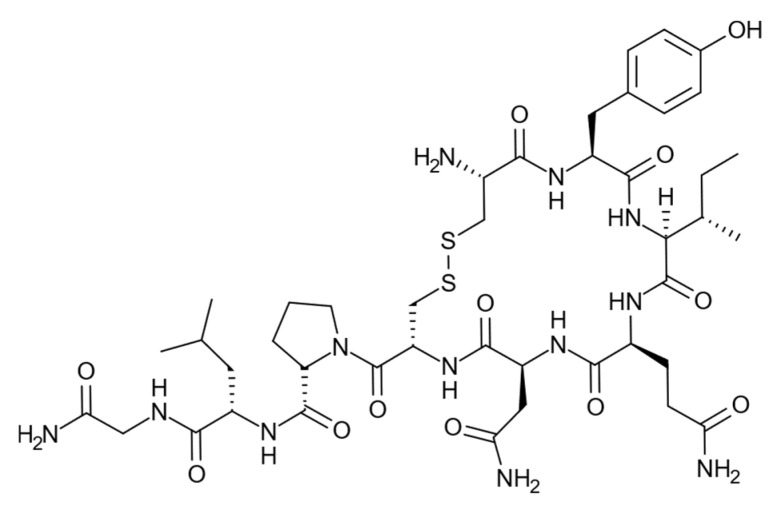
Oxytocin.

Generally, the protein templates used in studies reported to date have contained significant numbers of polar residues, many charged, complementary with the acrylamide and acrylate monomer combinations most often used [25]. In the present study, we have investigated the use of the neuropeptide oxytocin [26], Figure 1, as a template in nanoparticle synthesis using the template immobilized on glass beads. This peptide has only one charged functionality at physiological pH, the N-terminal primary amine, which may explain the difficulties in raising antibodies against this peptide [27,28]. As this N-terminal primary amine is used for amide coupling to the solid support, imprinted nanoparticle synthesis is performed without the presence of this charged functionality. Several studies have described attempts to imprint oxytocin using either monoliths or thin film formats. [29,30,31,32,33,34].

**Figure 2:**
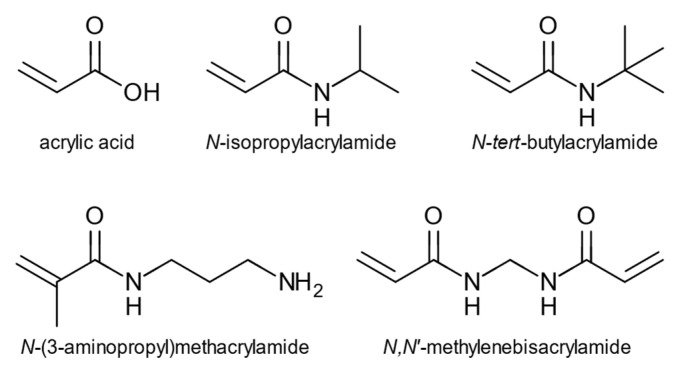
Monomers used for the synthesis of oxytocin-molecularly imprinted nanoparticles.

The nanoparticles were prepared by high dilution polymerization using a monomer cocktail previously found useful for high affinity protein targeted nanoparticles [35]. This was comprised of *N*-isopropylacrylamide (NIPAM), *N*-*t*-butylacrylamide (TBAM), acrylic acid (AA), and aminopropylmethacrylamide hydrochloride (APMA) and *N*,*N*′-methylenebisacrylamide (BIS) as a cross-linking monomer, Figure 2. NMR- and molecular dynamics studies were used to investigate monomer involvement in template recognition, and the recognition of oxytocin by the oxytocin imprinted nanoparticles (O-NPs) was investigated using a quartz crystal microbalance (QCM) using oxytocin-immobilized and control surfaces.

## 2. Results and Discussion

The polymer composition used for synthesis of O-NPs was adopted from previous studies where similar mixtures of polar and hydrophobic monomers were successfully used for imprinting of peptides and proteins [17,21]. To investigate the extent of interactions between the monomers and oxytocin, ^1^H-NMR titration studies were undertaken. Increasing monomer concentrations resulted in chemical shift changes for oxytocin’s protons (Figures S2, S4, S6, S8 and S10). This was most pronounced for the polar monomers AA (Figure S2, Table S1) and APMA (Figure S4, Table S3), particularly for the amide proton of isoleucine. Nonlinear curve fitting resulted in apparent dissociation constants ranging from 0.4 to 10 mM for AA (Figure S3, Table S2) and 1 to 40 mM for APMA (Figure S5, Table S4). BIS, NIPAM and TBAM had much less influence on the NMR spectra of oxytocin (Tables S5–S7) and curve fitting failed, producing only unstable results. To gain further insight into pre-polymerization interactions, molecular dynamics studies were performed for a system mimicking the mixture used to synthesize O-NPs (Figure S12). In addition to providing further support for the interactions with AA and APMA observed using NMR, hydrogen bond- (Tables S8 and S9), radial distribution function (RDF)- and grid density analyses (Figure S13) also revealed considerable interactions between oxytocin and the non-polar components NIPAM, TBAM and BIS. Oxytocin imprinted nanoparticles were synthesized using a solid phase synthesis method [23]. The solid-phase methodology allows for facile separation of the O-NPs from the template, which is often considered the bottleneck in MIP synthesis protocols [36]. Glass beads were used as a solid support and were functionalized with a primary amine group. Glutaraldehyde was used as a cross-linker to attach the oxytocin template. Finally, a reduction step with sodium cyanoborohydride was performed to convert the formed unstable Schiff bases into secondary amines. Glass beads with immobilized oxytocin were dispersed into the monomer mixture, and polymerization was initiated using tetramethylethane-1,2-diamine (TEMED) as the catalyst and ammonium persulfate (APS) as the initiator. The resultant microbeads-MIP conjugates were washed several times with water at room temperature to remove all non-reacted monomers and low affinity imprinted nanoparticles. Tightly bound O-NPs were eluted using hot ethanol, concentrated, and purified by dialysis against water yielding an average of 1.8 mg (Table S10).

**Figure 3:**
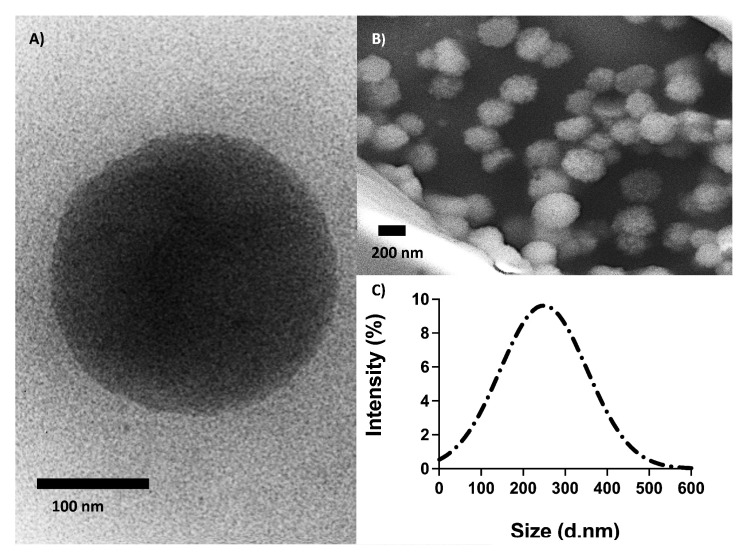
(**A**) Transmission electron- and (**B**) scanning electron micrographs, and (**C**) DLS size distribution of oxytocin imprinted nanoparticles.

The size and shape of the O-NPs were assessed using transmission electron microscopy (TEM), scanning electron microscopy (SEM) and dynamic light scattering (DLS) analysis (Figure 3). The diameter and surface charge were measured using DLS in water resulting in a Z-average of 248 ± 21 nm, a polydispersity index of 0.28 ± 0.019 and a zeta potential (ZP) of −0.086 mV. The TEM- and SEM images showed that the O-NPs were spherical with an average size of 230 nm. Together, TEM, SEM and DLS analyses demonstrated that the O-NPs had well-defined spherical structures and were homogenous in size.

**Figure 4:**
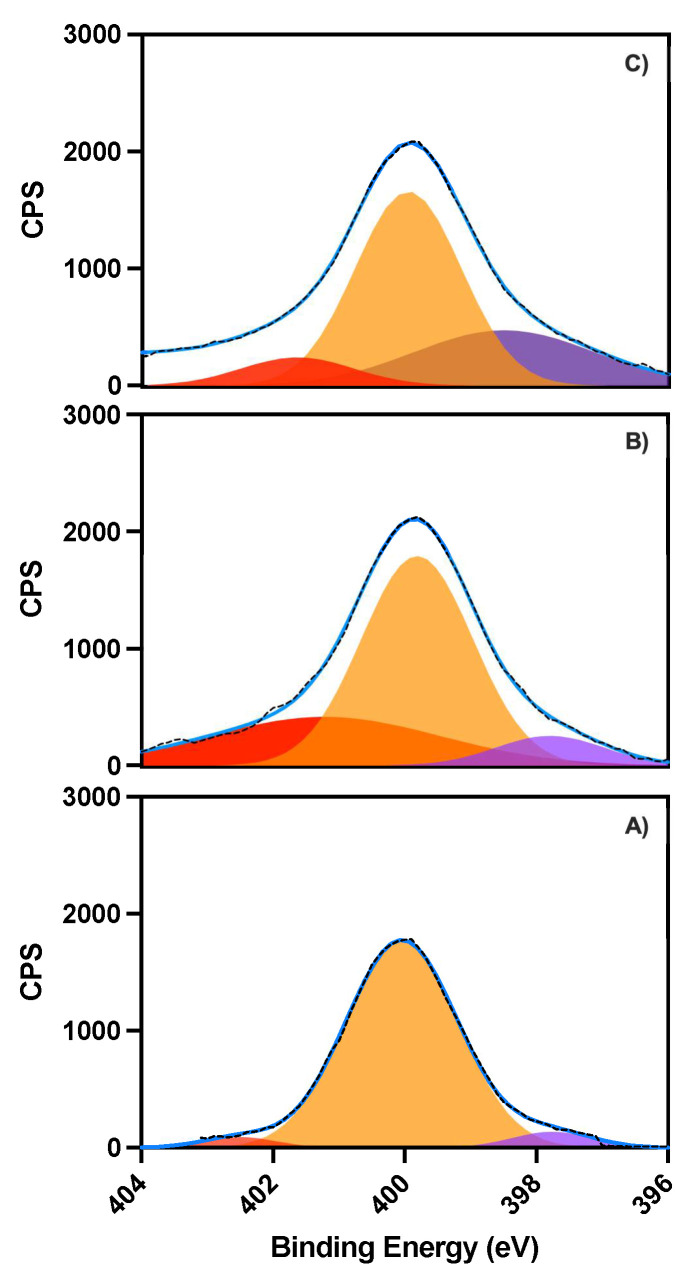
High resolution N1s XPS spectra; (**A**) ACA, (**B**) oxytocin-ACA, (**C**) O-NPs -oxytocin-ACA (CPS, counts per second).

X-ray photoelectron spectroscopy (XPS) measurements were performed to ascertain the oxytocin and nanoparticle binding process. Survey spectra of 6-aminocaproic acid (ACA) modified low non-specific binding (LNB) chips, recorded before and after oxytocin immobilization and after binding with O-NPs (Figure S14), shows respective bands for the anticipated elements, such as C1s, O1s and N1s around 286, 533 and 400 eV, respectively. Deconvolution of the N1s band in high resolution XPS spectra shows how the individual contributions from the different nitrogen moieties (-NH_2_, -NH-, and -NR_2_ at 397.6, 399.9 and 402.5 eV) change upon oxytocin immobilization (Figure 4B) and after binding of O-NPs (Figure 4C). The immobilization and binding are also reflected in the corresponding changes of the C1s band (Table S11) and by reflection–absorption IR spectroscopy (RAIRS) measurements with increasing intensities of the amide (1656 and 1536 cm^−1^) and C-H bands (2920 cm^−1^), relative to the carbonyl band of ACA (1710 cm^−1^), Figure S15.

**Figure 5:**
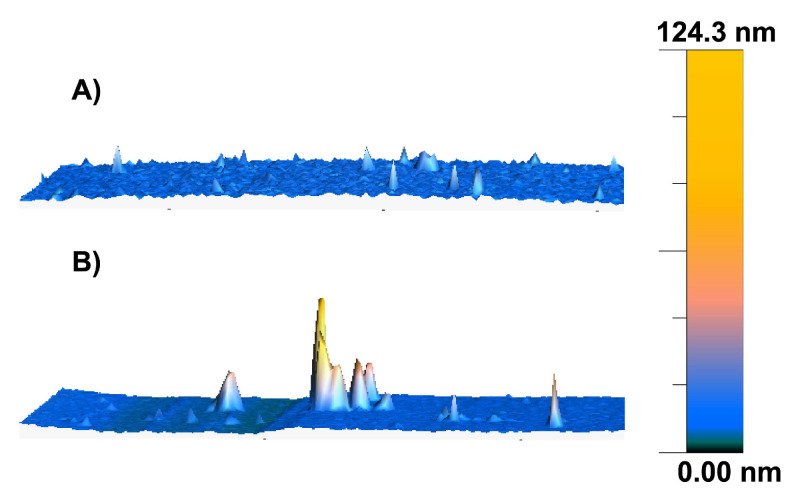
Surface topography of the oxytocin immobilized ACA functionalized gold surface (**A**) before and (**B**) after binding with O-NPs, mapped using AFM in non-contact mode (Image size 10 × 10 µm).

Functionalized gold surfaces were characterized by atomic force microscopy (AFM) analysis, which was conducted before and after binding of O-NPs on oxytocin-ACA functionalized gold surfaces. Figure 5 shows the transformation in roughness of the surface topography after the addition of the O-NPs, indicating the presence of nanoparticles on the oxytocin immobilized surface.

**Figure 6:**
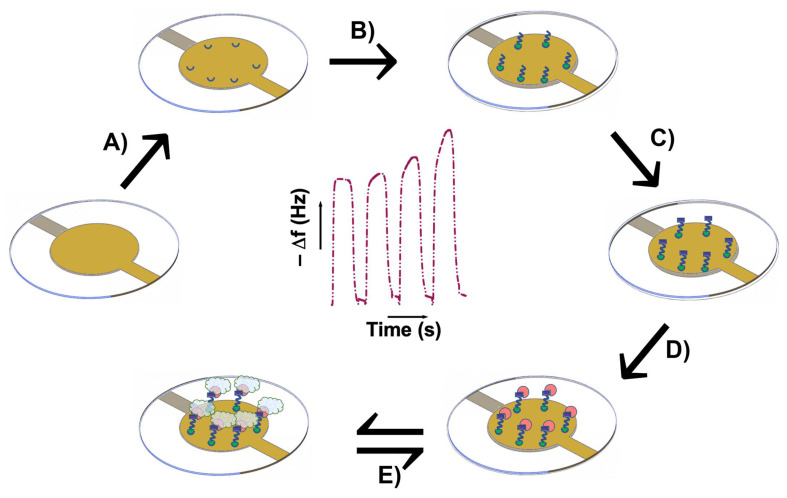
Schematic representation of the immobilization of oxytocin on QCM chips for the detection of O-NPs; (**A**) LNB carboxyl surface activation by EDC/sulfo-NHS, (**B**) ACA immobilization, (**C**) ACA surface activation by EDC/sulfo-NHS, (**D**) oxytocin immobilization, (**E**) O-NPs binding.

**Figure 7:**
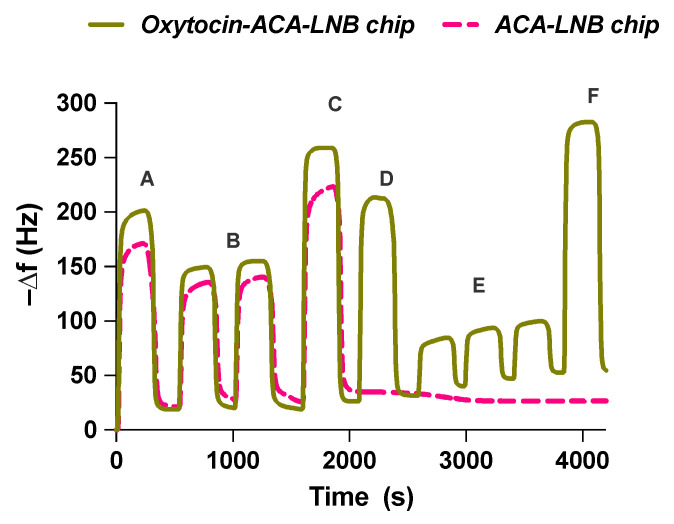
QCM trace during immobilization of oxytocin on LNB carboxyl chip. (**A**) Carboxyl activation of LNB-chip by EDC/Sulfo-NHS; (**B**) ACA immobilization (2 injections); (**C**) deactivation by ethanolamine; (**D**) ACA activation by EDC/Sulfo-NHS; (**E**) oxytocin immobilization (three injections); (**F**) deactivation by ethanolamine.

Oxytocin was immobilized on QCM chips as shown in Figure 6. The QCM surfaces were first functionalized with ACA as a spacer to localize oxytocin at a similar distance from the surface as during O-NP synthesis. Oxytocin was then attached to ACA using 1-ethyl-3-(3-dimethylaminopropyl)carbodiimide (EDC)/*N*-hydroxysulfosuccinimide (sulfo-NHS) coupling chemistry, followed by deactivation with ethanolamine. The process was monitored throughout the change in resonant frequency, Figure 7. This allowed the estimation of the amount of oxytocin immobilized on the sensor surface (14 ± 4 ng). Control (without peptide) and reference (vasopressin, Figure S14) chips were similarly prepared.

**Figure 8:**
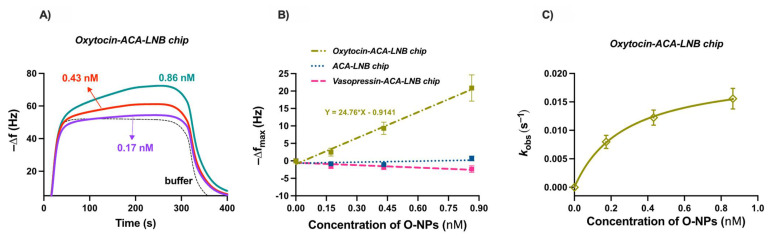
(**A**) Resonant frequency changes observed for repeated injections of O-NPs on oxytocin functionalized LNB-carboxyl QCM chip, *n* = 8; (**B**) linear calibration curve for O-NPs binding to different modified surfaces, maximum change in resonant frequency as a function of concentration of O-NPs; (**C**) variation of *k*_obs_ constant against concentrations of O-NPs to oxytocin surface. The *k*_obs_ values were calculated using nonlinear curve fitting from the association part of the QCM binding curves.

The functionalized QCM chips were used to evaluate the binding of O-NPs to oxytocin. The oxytocin and reference surfaces were allowed to stabilize in water followed by injections of buffer (10 mM HEPES, 150 mM NaCl, 0.005% Tween^®^20, pH 7.4) or different concentrations of O-NPs, ranging from 0.17 nM to 0.86 nM. Injection resulted in a concentration dependent frequency change, Figure 8A. After 300 s, elution of the O-NPs was facilitated by switching the mobile phase to water. Injections of buffer were used to allow for subtraction of bulk effects. The maximum resonant frequency changes, ranging from 4 to 22 Hz, varied linearly with the concentration of O-NPs, Figure 8B. The slope of the resulting line is indicative of the sensitivity of the system and was found to be 24.76 Hz/nM (R^2^ = 0.9933). To investigate the selectivity, control and reference chips were interrogated with O-NPs. The O-NPs did not show affinity for either the ACA or vasopressin modified surfaces. The low affinity of the O-NPs for the carboxylate bearing ACA surfaces indicates that the observed O-NP–oxytocin–surface interaction is not driven by non-oxytocin related interactions. This is further supported by the low affinity of the O-NPs for the vasopressin immobilized surface. The NMR data indicated a relatively strong interaction between functional monomers and the isoleucine residue of oxytocin, and accordingly tighter interaction in the resultant polymers. This residue is a point of difference between these peptides, where, in the case of vasopressin, a bulkier phenylalanine is present. Moreover, the low affinity of the effectively neutral charged O-NPs for the positively charged arginine sidechain-bearing vasopressin highlights the importance of the unique structure and functionality of the template and immobilized oxytocin moiety for O-NP capture. The affinity of the O-NPs for the oxytocin surface was quantified as previously described [37]. The apparent rate constants *k*_obs_ were deduced by fitting the initial parts of the frequency response curves to Equation S4. The determined *k*_obs_ values (R^2^ = 0.99–0.9993) varied nonlinearly with the concentration of the O-NPs, Figure 8C. The binding affinity of the O-NPs for the peptide surface is reflected in the determined apparent dissociation constant, *k_d_* ≈ 0.3 ± 0.02 nM (standard error of the mean, R^2^ = 0.9995). This is an order of magnitude of the binding weaker than observed for protein imprinted NPs, where the larger template offers additional interaction points. Most interestingly, the observed binding affinity is comparable to that of commercially available polyclonal oxytocin antibodies, 0.02–0.2 nM [38]. This compares favourably with previous attempts to produce oxytocin molecularly imprinted materials. The affinity is significantly better than was observed for monolith-derived polymers (47–102 μM, [29]) and comparable to binding observed using oxytocin imprinted thin films (0.03–11 nM, [33,34]). This highlights the potential for using oxytocin imprinted nanoparticles as substitutes for antibodies in diagnostic applications.

## 3. Materials and Methods

### 3.1. Materials

Spheriglass A glass beads with an average diameter of 70–90 µm were from Potters Industries LLC., Malvern, PA, USA; sodium hydroxide was from AppliChem, Darmstadt, Germany; sulphuric acid (95–97%), hydrogen peroxide (30%), BIS, NIPAM, TBAM, AA, APMA, APS, TEMED, ACA, 1,2-bis(triethoxysilyl)ethane (30%) (BTSE), 11-mercaptoundecanoic acid (MUDA), phosphate buffered saline (PBS) (10 mM, pH 7.2), sodium cyanoborohydride, gold coated silicon wafer (99.999% (Au), layer thickness 1000 Å, 99.99% (Ti)) and deuterium oxide (D_2_O, containing 0.75 wt. % 3-(trimethylsilyl)propionic-2,2,3,3-*d*_4_ acid sodium salt (TSP-*d*_4_)) were from Sigma-Aldrich, Steinheim, Germany; *N*-(6-aminohexyl)aminomethyltriethoxysilane (AHAMTES) was from abcr GmbH, Karlsruhe, Germany; dialysis tubing (SnakeSkin^TM^, 10,000 MWCO) was from Thermo Scientific, Rockford, IL, USA; solid-phase extraction (SPE) cartridges were from Agilent Technologies, Santa Clara, CA, USA (60 mL, 1–1/16 in, 20 µm); glutaraldehyde (25%) was from Merck-Eurolab, Stockholm, Sweden; acetic acid (glacial) and ammonia solution (25%) were from Merck KGaA, Darmstadt, Germany; absolute ethanol and acetone was from VWR International, Fontenay-sous-Bois, France; dry toluene was from Scharlau, Barcelona, Spain; oxytocin and vasopressin was from Prospec, Rehovot, Israel and LNB carboxyl modified gold QCM chips, HEPES buffer (100 mM HEPES, 1.50 M NaCl, 0.05% Tween^®^20, pH 7.4), ethanolamine (1 M, pH 8.5), 1-ethyl-3-(3-dimethylaminopropyl)carbodiimide (EDC, 0.4 mM) and *N*-hydroxysulfosuccinimide (sulfo-NHS, 0.1 mM) were from Attana AB, Stockholm, Sweden. The water used was of Milli-Q grade.

### 3.2. Molecular Dynamics Simulations

The AMBER 2018/AMBERTOOLS 2018 software suite [39] was used for setup, parameterization and simulation of systems except for water (structure and model available in the AMBER distribution) and oxytocin. A human oxytocin structure (PDB ID: 2MGO [40]) was obtained from the RCSB PDB database. This structure was stripped of hydrogen atoms and prepared for use with AMBER using the pdb4amber module. The N-terminus was capped with an acetyl group using the software AVOGADRO [41] (version 1.2.0) according to instructions available in the AMBER manual. The resulting structure file was checked using the pdb4amber module and xLEaP, producing a structure file compatible with the AMBER standard protein forcefield, protein.ff14SB. The other structures were generated using the AVOGADRO software, including initial optimization and energy minimization using the general amber forcefield (GAFF) [42]; ANTECHAMBER was used to assign partial atomic charges through the AM1-BCC charge method and derive parameter files [43,44], assigning GAFF2 (a development of GAFF) atom types [39,42,45].

Random initial starting configurations were generated using the PACKMOL software [46,47]. Simulation input was assembled using the xLEaP program and parameterized using GAFF2. Initial energy minimization was performed (50,000 steepest descent steps followed by 50,000 conjugate gradient steps). Temperature equilibration was then performed, heating from 0 to 323.15 K using Langevin dynamics with a collision frequency of 1.0 ps^−1^ over 500 ps (250,000 iterations with a 2 fs timestep) at constant volume. The pressure was then equilibrated using the Berendsen barostat and isotropic positional scaling with a pressure relaxation time of 2.0 ps for isothermal pressure equilibration to 1 bar at 323.15 K. Velocity resetting was performed every 1 ns using a random seed number to avoid energy aggregation. Equilibration was performed until stable values of temperature, energy, and pressure were obtained. Production phase simulation data were collected during 100 ns under conditions of constant volume and temperature (323.15 K) maintaining constant temperature using Langevin dynamics with a collision frequency of 1.0 ps^−1^ and implementing velocity resetting every 1 ns. Periodic boundary conditions and a 9 Å non-bonded interaction cut-off were used. Long-range electrostatics were treated using the particle mesh Ewald (PME) summation method [48,49]. Long range van der Waals interactions were treated using a continuum model correction. All bonds to hydrogen atoms were constrained using the SHAKE algorithm, allowing the set time step (2 fs). Simulation data from production phase calculations were analyzed using the CPPTRAJ module (included in AMBERTOOLS 2018) [50,51]. Hydrogen bond analyses were performed using the HBOND module applying default values for distance and angle cut-offs. RDF analyses were performed using the RADIAL module applying a bin-width of 0.1 Å and a maximum radius of 20 Å. Three-dimensional density maps were calculated using the GRID module with a 0.5 Å grid spacing, expanding out 20 bins in all directions with systems centered on all residues belonging to each separate oxytocin molecule using the AUTOIMAGE function of CPPTRAJ. The density maps were visualized using UCSF Chimera, developed by the Resource for Biocomputing, Visualization, and Informatics at the University of California, San Francisco (supported by NIH P41-GM103311) [52]. Further analyses and visualization was also conducted using the Visual Molecular Dynamics (VMD) software [53]. Data acquisition simulations and analyses were performed on resources provided by the Swedish National Infrastructure for Computing (SNIC) at AURORA, at RACKHAM and using resources provided by the Linnaeus University Centre for Data Intensive Sciences and Applications (DISA). 2D molecular structures were drawn using Marvin 20.19.0, 2020, ChemAxon (http://www.chemaxon.com, accessed on 31 January 2022).

### 3.3. Nuclear Magnetic Resonance (NMR)

^1^H-NMR spectra were collected at 298K for 1 mM solutions of oxytocin in the presence of increasing concentrations of BIS, AA, APMA, NIPAM, and TBAM using a Bruker Avance 400 MHz spectrometer (Bruker BioSpin AG, Fällanden, Switzerland) using the standard Bruker pulse sequence “zgesp” for water suppression (256 scans). The solvent used was H_2_O containing 10% (*v*/*v*) D_2_O with TSP-*d*_4_ as chemical shift reference (0 ppm). The chemical shifts of selected ^1^H-resonances were determined using the software package MestReNova v. 14.2.0 (Mestrelab Research SL, Santiago de Compostela, Spain). Changes in chemical shifts (Δδ) were plotted against concentration and fitted to a one-site interaction model (y = *B*_max_·X (*k_d_* + X)) using nonlinear fitting in the software package Prism v. 9.1.1 (GraphPad Software, San Diego, CA, USA).

### 3.4. Synthesis of Oxytocin-Molecularly Imprinted Nanoparticles (O-NPs)

#### 3.4.1. Glass Bead Activation

Glass beads (200 g) were boiled in aqueous sodium hydroxide (1 M, 0.8 mL of solution per g of glass beads) for 15 min and then rinsed with water (8 × 200 mL). The glass beads were then incubated for 60 min of 20% (*v*/*v*) sulfuric acid (80 mL), washed with water (8 × 200 mL) and acetone (3 × 200 mL), and dried at 120 °C for 6 h.

#### 3.4.2. Silanization

Activated glass beads (200 g) were incubated for 12 h at 80 °C under reflux in a solution (80 mL) of 3.7% (*v*/*v*) AHAMTES and 0.12% (*v*/*v*) BTSE in dry toluene to obtain amine bearing beads. Next, the beads were washed with toluene (3 × 200 mL), ethanol (3 × 200 mL), water (3 × 200 mL), acetone (3 × 200 mL) and then dried at 150 °C for 2 h.

#### 3.4.3. Template Immobilization

To immobilize oxytocin, silanized glass beads (30 g) were incubated in 12 mL of 7% (*v*/*v*) glutaraldehyde prepared in PBS (10 mM, pH 7.2) for 2 h. After washing with water (3 × 200 mL) and PBS (3 × 200 mL), the glass beads were incubated in 25 mL of a solution of oxytocin (0.5 mg/mL) in PBS (10 mM, pH 7.2) for 2 h. The glass beads were rinsed with water (3 × 200 mL) and PBS (3 × 200 mL). The oxytocin-derivatized glass beads were then incubated in 25 mL of a sodium cyanoborohydride aqueous solution (1 mg/mL) for 30 min. The derivatized glass beads were washed with water (3 × 200 mL) and then PBS (3 × 200 mL) and dried for 30 min under vacuum.

#### 3.4.4. Polymer Synthesis

Aqueous solid-phase synthesis was used for the preparation of O-NPs. In a typical synthesis, the polymerization mixture was composed of NIPAM (39 mg, 0.345 mmol), BIS (6 mg, 0.0388 mmol), TBAM (33 mg, 0.259 mmol, dissolved in1 mL of ethanol), AA (2.2 μL, 0.0321 mmol), and APMA (5.8 mg, 0.0325 mmol). The monomers were dissolved in 99 mL Milli-Q, 50 mL of which was mixed with 30 g of oxytocin-derivatized glass beads. This mixture was sonicated for 5 min and then purged with nitrogen for 30 min. Polymerization was initiated by the addition of APS (0.5 mL, 60 mg/mL) and TEMED (1 mL, 30 μL/mL) and was carried out at room temperature for 1 h. The beads were then washed with water (10 × 20 mL) at 22 °C in an SPE cartridge to remove unreacted materials and low affinity O-NPs. High-affinity O-NPs were eluted with hot ethanol (65 °C, 10 × 20 mL). The combined eluates were concentrated to 20 mL using a rotary evaporator and then dialyzed against water for 72 h, changing the water every 12 h. The yield of the O-NPs was determined based on a calibration curve obtained by evaporating the O-NPs solution and weighing dry sample (Figure S1), and the apparent molarities of O-NPs were calculated as described by Hoshino et al. (Equation (S1)) [17].

### 3.5. Characterization of O-NPs

#### 3.5.1. Dynamic Light Scattering (DLS) and Zeta Potential (ZP)

Surface charge and particle size analyses of the O-NPs were performed using a Zetasizer Nano ZS (He-Ne laser; Malvern Instruments Ltd., Worcestershire, UK) furnished with a back-scattering detector (173°) at 25 °C. 100 µL (95 µg/mL) of O-NPs were dispersed in 900 µL of water, sonicated for 15 s using a Branson SFX 150 digital sonifier equipped with a Branson 4C15 40 kHz converter (50% intensity) (Process Equipment & Supply, Inc., North Olmsted, OH, USA). Triplicate measurements were performed in a pre-rinsed dip cell in automatic mode.

#### 3.5.2. Scanning Electron Microscopy (SEM)

SEM images were obtained using a LEO Ultra 55 instrument (Carl Zeiss AG, Oberkochen, Germany) equipped with a field emission electron gun. A diluted solution of O-NPs was deposited on Si-wafers and placed on black carbon tape attached to alumina stubs and sputtered with a thin layer of palladium using a LEICA EM SCD 500 sputtering unit (Leica Microsystems GmbH, Wetzlar, Germany) before being inserted in the SEM instrument. The vacuum level in the sample chamber was maintained at 10^−5^ mbar. A 3 kV potential was applied to the electron gun to generate the electron beam used to scan the samples.

#### 3.5.3. Transmission Electron Microscopy (TEM)

TEM images were obtained using a TECNAI G2 Spirit instrument (FEI Company, Hillsboro, OR, USA) with an acceleration voltage of 80 kV. The TEM samples were prepared by drop coating a dilute solution of particles on copper grids of 100 mesh coated with carbon.

#### 3.5.4. Immobilization of Oxytocin on QCM Chips

A LNB carboxyl chip was inserted into the QCM sensor system (Cell-200, Attana AB, Stockholm, Sweden) and allowed to stabilize at a flow rate of 100 μL/min in running buffer (10 mM HEPES, 150 mM NaCl, 0.005% Tween^®^20, pH 7.4). When the baseline was stable (drift < 0.2 Hz/min), the flow rate was lowered to 10 μL/min. The chip surface was activated by injecting 50 μL of a 1:1 mixture of EDC and sulfo-NHS. After 10 min, the loops were rinsed with water (2 × 200 μL) and running buffer (3 × 200 μL). 2 × 50 μL ACA (100 mM in running buffer) was injected over the activated chip surface. The loops were rinsed again as above. To deactivate any remaining NHS esters, 50 μL of 1 M ethanolamine pH 8.5 were injected and, after 10 min, the loops were rinsed as above. To couple oxytocin to the ACA-LNB chip, the surface was activated by injecting 50 μL of a 1:1 mixture of EDC and sulfo-NHS, 3 × 50 μL oxytocin (1 mM in running buffer) and 50 μL of 1 M ethanolamine pH 8.5. The loops were rinsed between each step. The amount of immobilized oxytocin was determined using the manufacturer’s Sauerbrey equation based protocol, where the total mass of oxytocin immobilized is given by the change of frequency multiplied by 0.7 ng (mass corresponding to 1 Hz of frequency shift) [54].

#### 3.5.5. O-NP-QCM Binding Studies

O-NPs (900 μL, 95 µg/mL) were dispersed in 100 μL of HEPES buffer (100 mM HEPES, 1.50 M NaCl, 0.05% Tween^®^20, pH 7.4), probe sonicated for 15 s, and then further diluted in 10 mM HEPES buffer (10 mM HEPES, 150 mM NaCl, 0.005% Tween^®^20, pH 7.4) at a series of concentrations. The flow rate was set to 10 µL/min and O-NPs were allowed to flow over an oxytocin modified QCM chip for 300 s before switching to water for regeneration of the chip surface. The binding data were analyzed using the software packages Origin v. 6.1 (OriginLab Corporation, Northampton, MA, USA) and Prism v. 9.1.1 (GraphPad Software, San Diego, CA, USA).

#### 3.5.6. X-ray Photoelectron Spectroscopy (XPS)

XPS samples were prepared as above on QCM LNB chips and spectra were acquired using an AXIS-Supra XPS instrument (Kratos Analytical, Manchester, UK) using the selected area analysis mode with a nominal area of analysis of 500 μm and monochromated Al KαX-rays producing photons of 1486 eV energy. The anode was operated at a power of 90 W. Wide scans (step size 1eV, pass energy 200 eV, dwell time 100 ms, range 136 to 1491 eV) and narrow scans (0.100 eV step size, pass energy 200 eV, 100 ms dwell time) of the N 1s region were acquired from four separate areas on each sample. Data analysis and curve fitting were performed using Origin v. 6.1 (OriginLab Corporation, Northampton, MA, USA).

#### 3.5.7. Reflection–Absorption IR Spectroscopy (RAIRS)

##### Surface Cleaning

Gold coated microslides (2 × 4 cm), procured from Linköping University, were cleaned by soaking in 40 mL piranha solution (H_2_O_2_:H_2_SO_4_, 1:3, *v*/*v*) for 1 min (*Caution: piranha solution reacts violently with organic compounds and is dangerous when in contact with skin or eyes*), washed with water (5 × 40 mL), and dried with a stream of N_2_ gas (UHP grade). Next, the slides were immersed in 35 mL base piranha solution (water:H_2_O_2_:25% NH_3_, 5:1:1) at 80 °C for 3 min (*Caution: base piranha solution is dangerous when in contact with skin or eyes and should be handled carefully*), rinsed under a stream of water (10 s on the gold side, 5 s on the back side, 5 s on gold side again), and dried under N_2_. The gold surfaces were immediately used for further modification.

##### Preparation of Self-Assembled Monolayers (SAMs)

SAMs were prepared by immersing the cleaned gold surfaces in 10 mL of thiol monomer solution (1 mM MUDA in absolute ethanol:acetic acid, 9:1) for 12 h at 22 °C in the dark. Then, the gold surfaces were rinsed 3 times each in absolute ethanol:acetic acid, 9:1, ethanol and water, dried under a stream of N_2_ and stored under inert conditions until further modification.

##### Oxytocin Immobilization and O-NP Binding

Oxytocin was coupled to the MUDA modified gold slides using the same chemistry as above for the QCM chips. Slides were sequentially immersed in sulfo-NHS:EDC, 1:1 for 15 min, 100 mM ACA in HEPES (10 mM HEPES, 150 mM NaCl, 0.005% Tween^®^20, pH 7.4) for 30 min, 1 M ethanolamine for 15 min, sulfo-NHS:EDC, 1:1 for 15 min, 1 mM oxytocin in HEPES (10 mM HEPES, 150 mM NaCl, 0.005% Tween^®^20, pH 7.4) for 30 min and 1 M ethanolamine for 15 min, with thorough rinsing in water between each step. Oxytocin modified slides were finally immersed in a solution containing 1 mL of O-NPs (95 µg/mL) dispersed in 4 mL water for 30 min followed by drying with UHP grade N_2_.

##### RAIRS Measurements

Infrared spectra were acquired on a Bruker Vertex 70 spectrometer (Bruker Optics GmbH & Co. KG, Ettlingen, Germany) using a grazing angle (85°) reflection setup, equipped with an LN2 cooled MCT detector and continuous N_2_ purging. All spectra were acquired at 2 cm^−1^ resolution between 4500 and 600 cm^−1^, as a summation of 200 scans, while a deuterated hexadecanethiol SAM on an Au surface was used as a reference. A three-term Blackmann–Harris apodization function was applied to the interferograms, prior to the Fourier transformation. The measurement chamber was maintained under an inert atmosphere throughout experiments by purging with nitrogen gas at positive pressure.

#### 3.5.8. Atomic Force Microscopy (AFM)

AFM measurements were performed using a Dimension 3100 SPM from Veeco Instruments Inc. (Plainview, NY, USA) to evaluate the topographical features of the oxytocin-gold surfaces in the non-contact mode, before and after binding of O-NPs. In this mode, the silicon probes were allowed to oscillate at their resonant frequency, 339 kHz, for imaging the surface. The scan rate was 1 Hz, and the images were obtained in 10 × 10 μm size. All measurements were performed at ambient temperature.

## 4. Conclusions

Oxytocin imprinted polymer nanoparticles were synthesized by glass bead supported solid phase synthesis and shown to have sub-nanomolar affinity for the immobilized template coated QCM studies, *k_d_* ≈ 0.3 ± 0.02 nM, comparable to those of commercial polyclonal antibodies. NMR and molecular dynamics studies were used to identify preferred monomer-template interactions, which was reflected in the preferential binding to the template relative to a structural analog, vasopressin. The high selectivity and affinity of the oxytocin imprinted nanoparticles opens up their use in developing bioassays for this important neuropeptide.

## Data Availability

Not applicable.

## Supplementary Materials

The supporting information can be downloaded at: https://www.mdpi.com/article/10.3390/ijms23052534/s1.
