# Supplementary material for: Oxytocin-Selective Nanogel Antibody Mimics
Source: Int J Mol Sci. 2022 Feb 25;23(5):2534. doi: 10.3390/ijms23052534 (PMC8909970; doi:10.3390/ijms23052534)
Supplement: Supplementary file 1 [file ijms-23-02534-s001.zip › ijms-1599279-Supplementary.pdf]

Oxytocin-selective nanogel antibody mimics

Rashmi Mahajan[1], Subramanian Suriyanarayanan[1,*], Gustaf D. Olsson[1], Jesper G. Wiklander[1], Teodor Aastrup[2], Börje Sellergren[3], Ian A. Nicholls[1,*]

[1] Linnaeus University Centre for Biomaterials Chemistry, Bioorganic and Biophysical Chemistry Laboratory, Department of Chemistry and Biomedical Sciences, Linnaeus University, SE-39182 Kalmar, Sweden; rashmi.mahajan@lnu.se (R.M.); gustaf.olsson@lnu.se (G.D.O.); jesper.wiklander@lnu.se (J.G.W.)

2 Attana AB, Greta Arwidssons väg 21, SE-11419 Stockholm, Sweden; teodor.aastrup@attana.com (T.A.)

3 Department of Biomedical Sciences and Biofilms-Research Center for Biointerfaces (BRCB), Faculty of Health and Society Malmö University SE-20506 Malmö, Sweden; borje.sellergren@mau.se (B.S.)

* Correspondence: esusu@lnu.se (S.S.); ian.nicholls@lnu.se (I.A.N.); Tel.: +46-480-446-200 (S.S. & I.A.N.)

Table of Contents

Ultraviolet–visible spectroscopy **2**

Nuclear magnetic resonance spectroscopy **3**

Molecular dynamics simulations **13**

Dynamic light scattering **15**

X-ray photoelectron spectroscopy **16**

Reflection adsorption infrared spectroscopy **17**

Quartz crystal microbalance studies **18**

Ultraviolet–visible spectroscopy

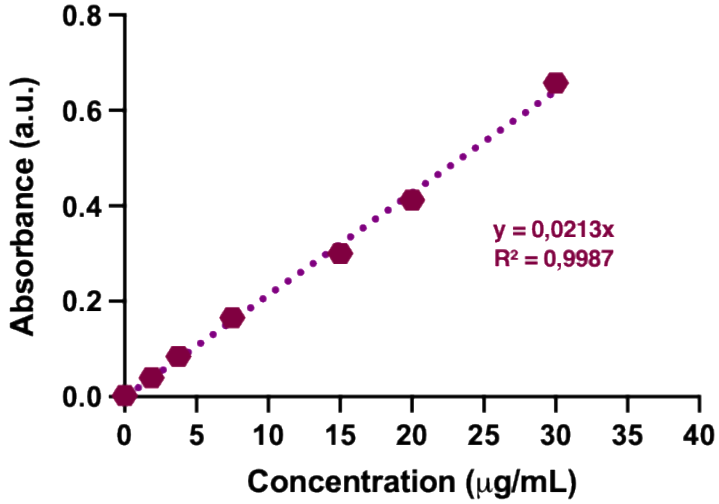

Figure S1. UV/Visible standard calibration curve of O-NPs

The apparent molarities of O-NPs were calculated using the following equation:

$$[M\ (app)] = \frac{6}{\pi N_A d^3 \rho} X \quad \text{(Equation S1)}$$

where N_A is Avogadro's constant, d is the hydrodynamic diameter of the particles (250 nm), ρ is polymer density (0.01 g cm^{-3} for swollen gel) and X is polymer concentration (mg ml^{-1}).

Nuclear magnetic resonance spectroscopy

a

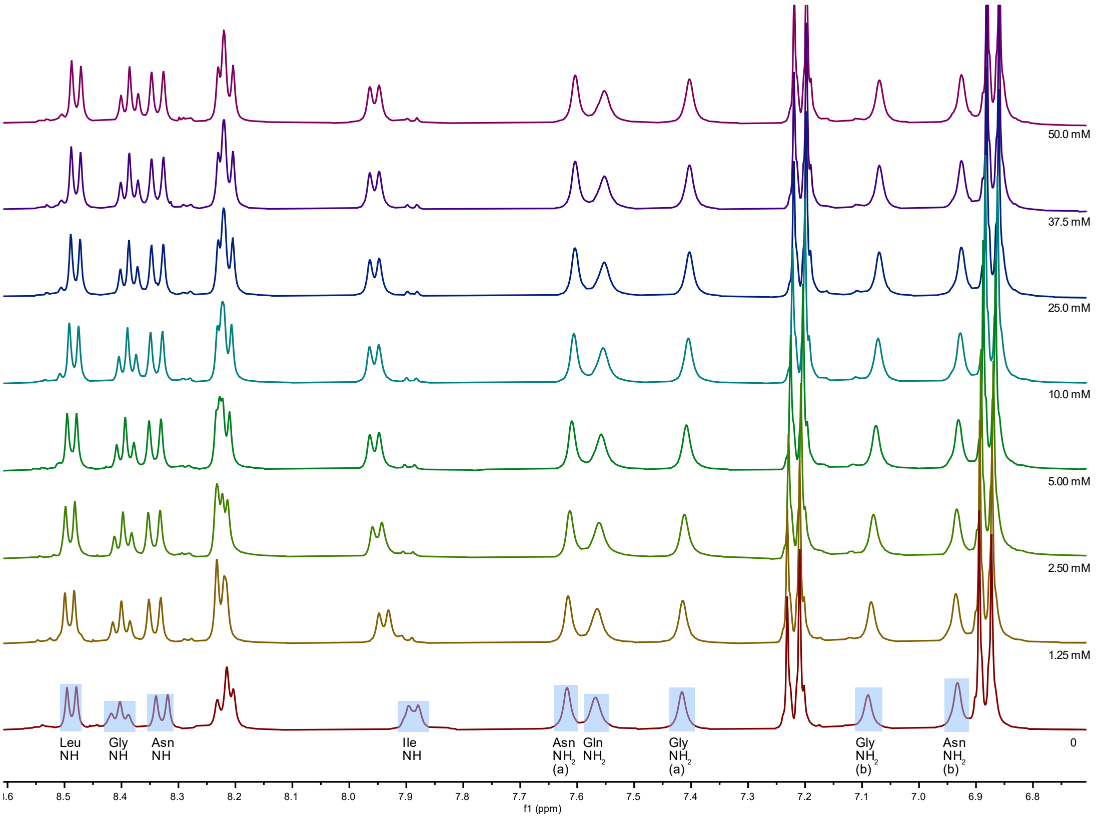

b

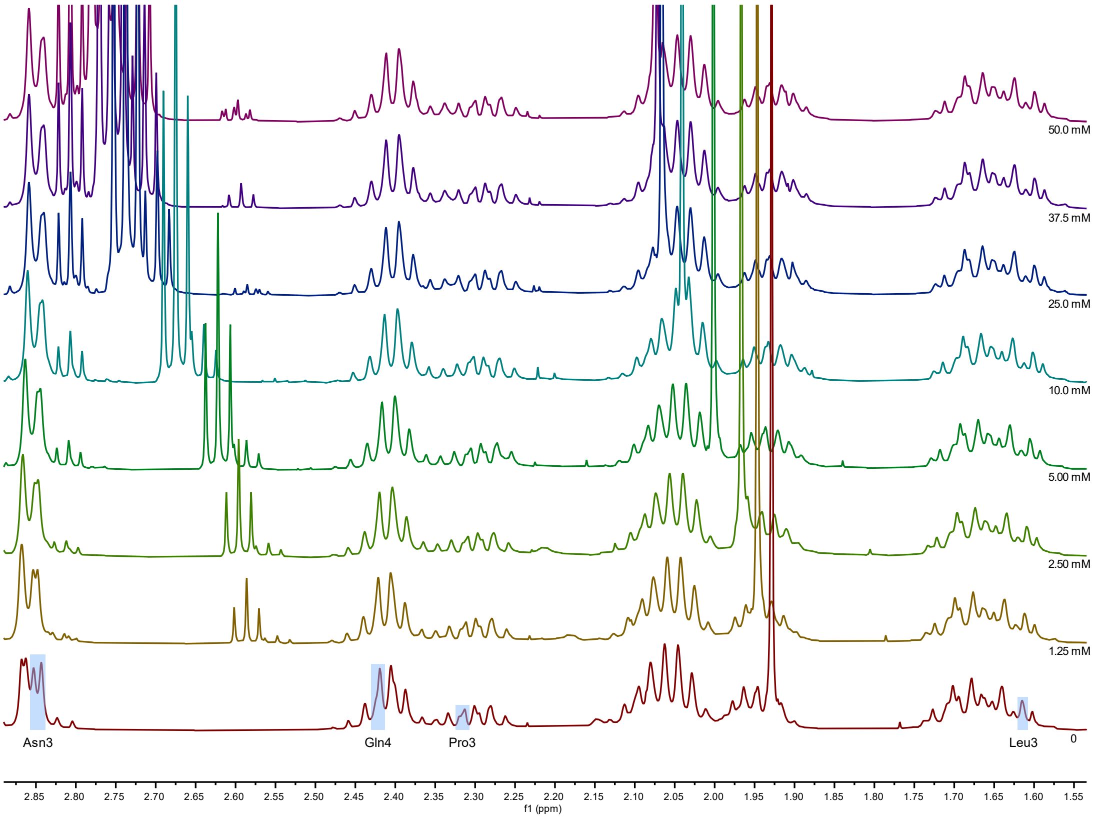

Figure S2. ^{1}H-NMR spectra of oxytocin in the presence of increasing concentrations of acrylic acid showing the amide (a) and aliphatic (b) regions with studied resonances indicated in blue.

Table S1. Chemical shifts and chemical shift changes of oxytocin proton resonances in the presence of increasing concentrations of acrylic acid (AA).

[AA] (M)	Chemical shift (ppm) Leu NH	Gly NH	Asn NH	Ile NH	Asn NH_2 (a)	Gln NH_2 (a)	Gly NH_2 (a)	Gly NH_2 (b)	Asn NH_2 (b)	Asn3	Gln4	Pro3	Leu3
0	8,4873	8,4029	8,3291	7,8875	7,6177	7,5679	7,4161	7,0895	6,9324	2,8481	2,4192	2,3131	1,6146
0,00125	8,4911	8,4001	8,3415	7,9396	7,6158	7,5651	7,4146	7,0837	6,9352	2,8507	2,4214	2,3114	1,6121
0,00250	8,4900	8,3973	8,3425	7,9510	7,6128	7,5617	7,4118	7,0798	6,9336	2,8494	2,4197	2,3090	1,6089
0,00500	8,4870	8,3933	8,3411	7,9559	7,6092	7,5580	7,4083	7,0755	6,9306	2,8467	2,4166	2,3052	1,6053
0,01001	8,4834	8,3895	8,3385	7,9563	7,6057	7,5546	7,4049	7,0718	6,9274	2,8432	2,4134	2,3019	1,6016
0,02501	8,4807	8,3869	8,3369	7,9558	7,6037	7,5526	7,4030	7,0697	6,9256	2,8419	2,4116	2,2999	1,5994
0,03752	8,4799	8,3862	8,3367	7,9557	7,6034	7,5525	7,4029	7,0695	6,9253	2,8416	2,4115	2,2997	1,5992
0,05003	8,4793	8,3857	8,3367	7,9559	7,6034	7,5524	7,4029	7,0695	6,9253	2,8416	2,4114	2,2996	1,5995

[AA] (M)	Absolute chemical shift change (ppm) Leu NH	Gly NH	Asn NH	Ile NH	Asn NH_2 (a)	Gln NH_2 (a)	Gly NH_2 (a)	Gly NH_2 (b)	Asn NH_2 (b)	Asn3	Gln4	Pro3	Leu3
0	0,0000	0,0000	0,0000	0,0000	0,0000	0,0000	0,0000	0,0000	0,0000	0,0000	0,0000	0,0000	0,0000
0,00125	0,0038	0,0028	0,0124	0,0521	0,0019	0,0028	0,0015	0,0058	0,0028	0,0026	0,0022	0,0017	0,0025
0,00250	0,0027	0,0056	0,0134	0,0635	0,0049	0,0062	0,0043	0,0097	0,0012	0,0013	0,0005	0,0041	0,0057
0,00500	0,0003	0,0096	0,0120	0,0684	0,0085	0,0099	0,0078	0,0140	0,0018	0,0014	0,0026	0,0079	0,0093
0,01001	0,0039	0,0134	0,0094	0,0688	0,0120	0,0133	0,0112	0,0177	0,0050	0,0049	0,0058	0,0112	0,0130
0,02501	0,0066	0,0160	0,0078	0,0683	0,0140	0,0153	0,0131	0,0198	0,0068	0,0062	0,0076	0,0132	0,0152
0,03752	0,0074	0,0167	0,0076	0,0682	0,0143	0,0154	0,0132	0,0200	0,0071	0,0065	0,0077	0,0134	0,0154
0,05003	0,0080	0,0172	0,0076	0,0684	0,0143	0,0155	0,0132	0,0200	0,0071	0,0065	0,0078	0,0135	0,0151

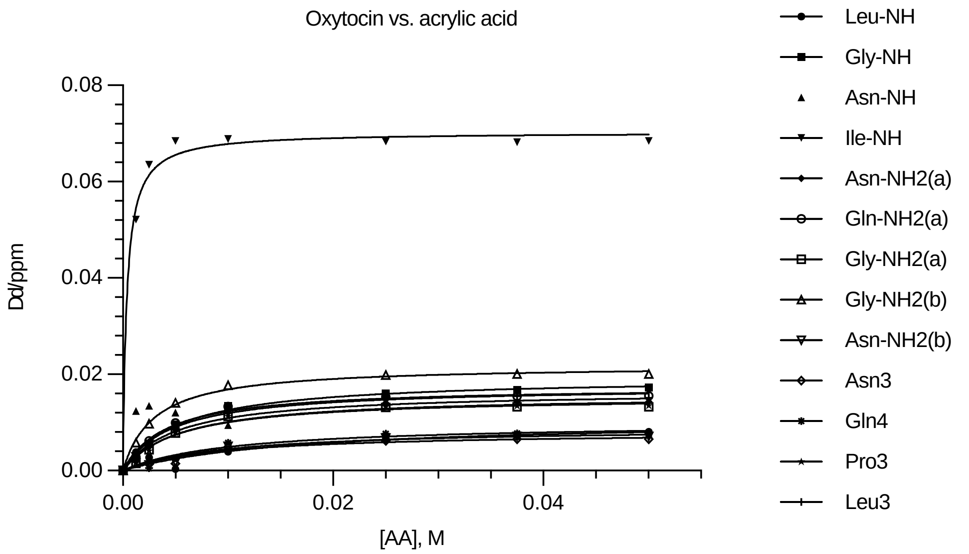

Figure S3. Plot of changes in chemical shift versus concentration of acrylic acid (AA). Curves correspond to the result of non-linear fitting.

Table S2. Results of non-linear fitting of changes in chemical shift to acrylic acid (AA) concentration.

	Best fit values B_{max}	K_d	95% CI B_{max}	K_d	Goodness of fit R^2
Leu-NH	0,01067	0,01675	0,00540 - 1,022·10^9	0,00139 - +infinity	0,760
Gly-NH	0,01938	0,00544	0,01816 - 0,02071	0,00429 - 0,00690	0,994
Asn-NH	0,00892	-0,00042	0,00708 - 0,01086	-0,00068 - -2,502·10^{-6}	0,850
Ile-NH	0,07026	0,00036	0,06769 - 0,07288	0,00022 - 0,00052	0,994
Asn-NH_2(a)	0,01650	0,00516	0,01484 - 0,01840	0,00349 - 0,00761	0,984
Gln-NH_2(a)	0,01747	0,00421	0,01604 - 0,01905	0,00302 - 0,00583	0,988
Gly-NH2(a)	0,01540	0,00539	0,01360 - 0,01753	0,00343 - 0,00847	0,978
Gly-NH_2(b)	0,02184	0,00298	0,02085 - 0,02287	0,00244 - 0,00361	0,995
Asn-NH_2(b)	0,00894	0,01001	0,00642 - 0,01445	0,00325 - 0,03468	0,904
Asn3	0,00810	0,00947	0,00568 - 0,01366	0,00275 - 0,03665	0,887
Gln4	0,00998	0,01040	0,00754 - 0,01452	0,00428 - 0,02816	0,934
Pro3	0,01568	0,00555	0,01396 - 0,01769	0,00365 - 0,00845	0,981
Leu3	0,01745	0,00466	0,01586 - 0,01924	0,00325 - 0,00666	0,986

a

b

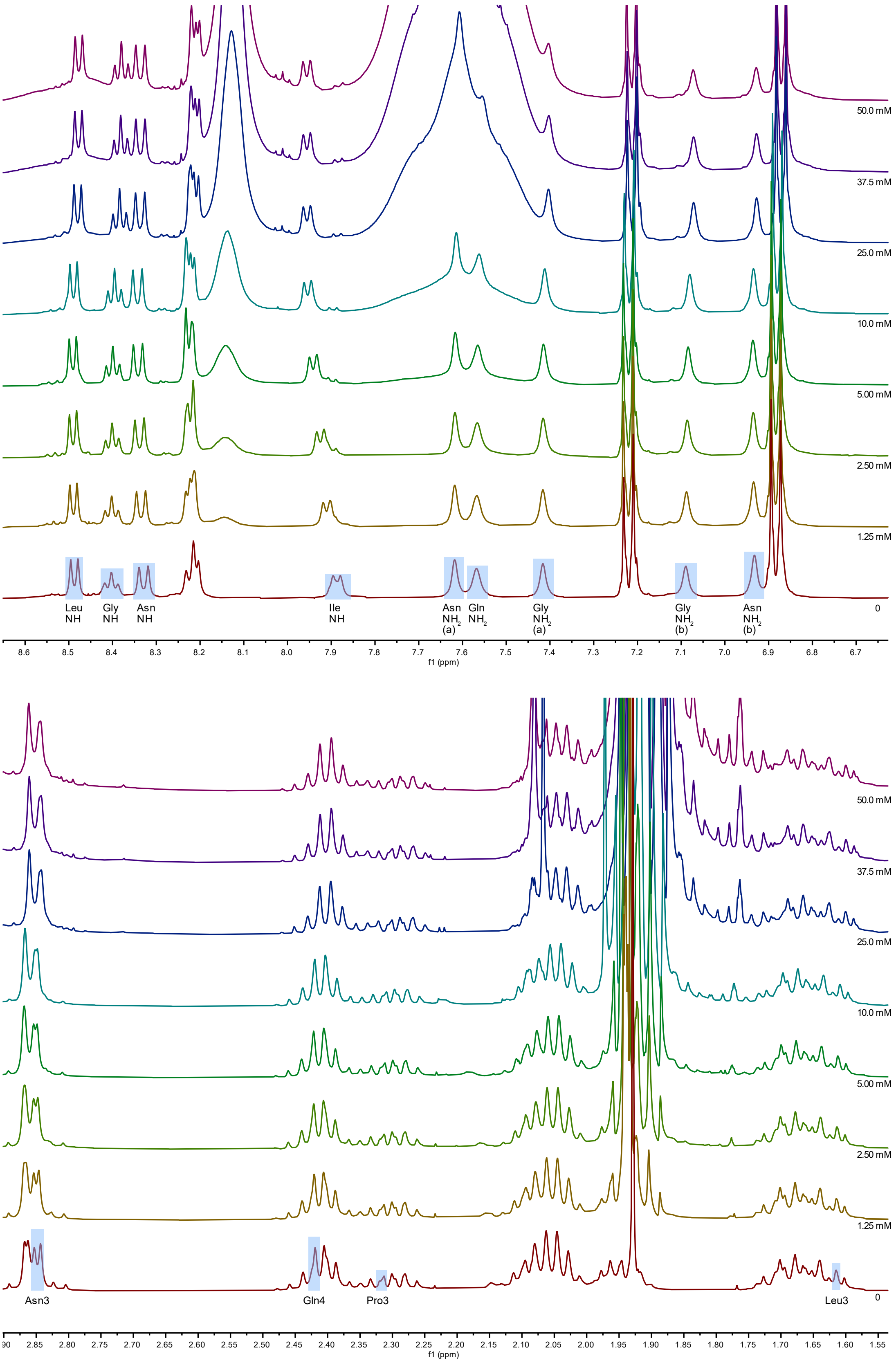

Figure S4. ^{1}H-NMR spectra of oxytocin in the presence of increasing concentrations of aminopropylmethacrylamide hydrochloride showing the amide (a) and aliphatic (b) regions with studied resonances indicated in blue.

Table S3. Chemical shifts and chemical shift changes of oxytocin proton resonances in the presence of increasing concentrations of aminopropylmethacrylamide hydrochloride (APMA).

[APMA] M	Chemical shift (ppm)												
	Leu NH	Gly NH	Asn NH	Ile NH	Asn NH_2 (a)	Gln NH_2 (a)	Gly NH_2 (a)	Gly NH_2 (b)	Asn NH_2 (b)	Asn3	Gln4	Pro3	Leu3
0	8,4873	8,4029	8,3291	7,8875	7,6177	7,5679	7,4161	7,0895	6,9324	2,8481	2,4192	2,3131	1,6146
0,00125	8,4894	8,4020	8,3350	7,9103	7,6176	7,5674	7,4160	7,0878	6,9343	2,8499	2,4208	2,3130	1,6145
0,00250	8,4904	8,4010	8,3384	7,9248	7,6169	7,5663	7,4155	7,0859	6,9351	2,8508	2,4215	2,3128	1,6134
0,00500	8,4910	8,3993	8,3419	7,9413	7,6163	7,5652	7,4146	7,0839	6,9358	2,8514	2,4216	2,3117	1,6119
0,00999	8,4893	8,3954	8,3430	7,9539	7,6140	7,5623	7,4120	7,0803	6,9346	2,8503	2,4199	2,3091	1,6090
0,02498	8,4798	8,3838	8,3369	7,9556	7,6070	7,5549	7,4033	7,0715	6,9274	2,8439	2,4122	2,3007	1,6003
0,03747	8,4781	8,3813	8,3363	7,9557	7,6076	0,0000	7,4028	7,0716	6,9276	2,8438	2,4116	2,3001	1,5995
0,04996	8,4772	8,3798	8,3364	7,9561	7,6101	0,0000	7,4035	7,0726	6,9283	2,8446	2,4118	2,3006	1,6000

[APMA] M	Absolute chemical shift change (ppm)												
	Leu NH	Gly NH	Asn NH	Ile NH	Asn NH_2 (a)	Gln NH_2 (a)	Gly NH_2 (a)	Gly NH_2 (b)	Asn NH_2 (b)	Asn3	Gln4	Pro3	Leu3
0	0,0000	0,0000	0,0000	0,0000	0,0000	0,0000	0,0000	0,0000	0,0000	0,0000	0,0000	0,0000	0,0000
0,00125	0,0021	0,0009	0,0059	0,0228	0,0001	0,0005	0,0001	0,0017	0,0019	0,0018	0,0016	0,0001	0,0001
0,00250	0,0031	0,0019	0,0093	0,0373	0,0008	0,0016	0,0006	0,0036	0,0027	0,0027	0,0023	0,0003	0,0012
0,00500	0,0037	0,0036	0,0128	0,0538	0,0014	0,0027	0,0015	0,0056	0,0034	0,0033	0,0024	0,0014	0,0027
0,00999	0,0020	0,0075	0,0139	0,0664	0,0037	0,0056	0,0041	0,0092	0,0022	0,0022	0,0007	0,0040	0,0056
0,02498	0,0075	0,0191	0,0078	0,0681	0,0107	0,0130	0,0128	0,0180	0,0050	0,0042	0,0070	0,0124	0,0143
0,03747	0,0092	0,0216	0,0072	0,0682	0,0101		0,0133	0,0179	0,0048	0,0043	0,0076	0,0130	0,0151
0,04996	0,0101	0,0231	0,0073	0,0686	0,0076		0,0126	0,0169	0,0041	0,0035	0,0074	0,0125	0,0146

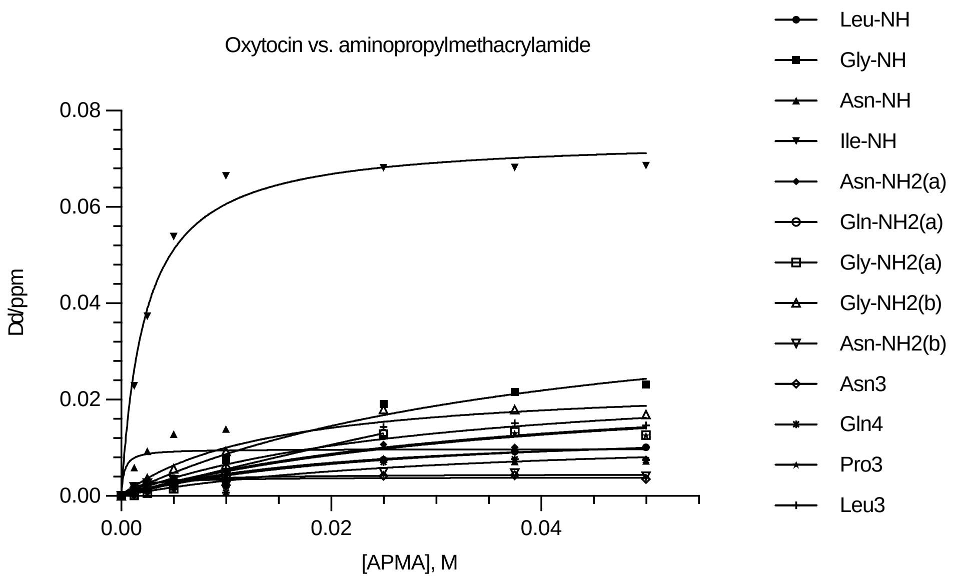

Figure S5. Plot of changes in chemical shift versus concentration of aminopropylmethacrylamide hydrochloride (APMA). Curves correspond to the result of non-linear fitting.

Table S4. Results of non-linear fitting of changes in chemical shift to aminopropylmethacrylamide hydrochloride (APMA) concentration.

	Best fit values		95% CI		Goodness of fit
	B_{max}	K_d	B_{max}	K_d	R^2
Leu-NH	0,01511	0,02555	0,0083 to 1,502E+09	0,00454 to +infinity	0,880
Gly-NH	0,04426	0,04092	0,0321 to 0,07865	0,02140 to 0,10160	0,985
Asn-NH	0,00973	0,00029	0,0064 to 0,01356	-0,00052 to 0,00273	0,597
Ile-NH	0,07443	0,00228	0,0690 to 0,08016	0,00160 to 0,00316	0,986
Asn-NH_2(a)	0,01414	0,02100	0,0081 to 0,08305	0,00491 to 0,31370	0,866
Gln-NH_2(a)	0,13090	0,22630	0,0647 to +infinity	0,10120 to +infinity	0,999
Gly-NH2(a)	0,02482	0,03675	0,0148 to 0,15830	0,01189 to 0,44730	0,939
Gly-NH_2(b)	0,02390	0,01382	0,0192 to 0,03175	0,00736 to 0,02795	0,971
Asn-NH_2(b)	0,00456	0,00214	0,0034 to 0,00627	0,00025 to 0,00883	0,820
Asn3	0,00391	0,00145	0,0030 to 0,00500	0,00016 to 0,00495	0,842
Gln4	0,01306	0,03107	0,0063 to +infinity	0,00434 to +infinity	0,837
Pro3	0,02518	0,03933	0,0148 to 0,20780	0,01269 to 0,61350	0,942
Leu3	0,02580	0,02950	0,0177 to 0,05572	0,01236 to 0,10650	0,961

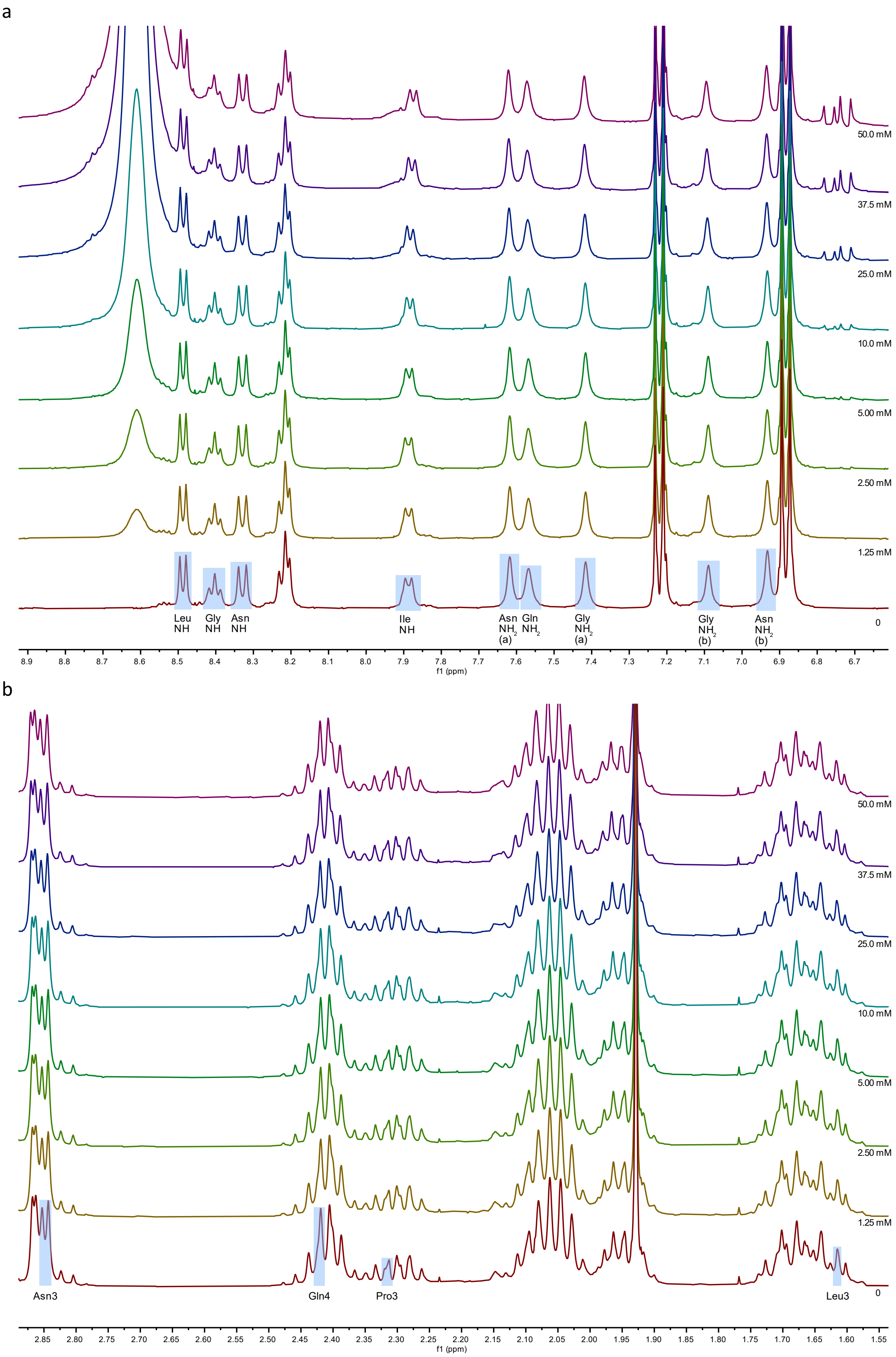

Figure S6. ^{1}H-NMR spectra of oxytocin in the presence of increasing concentrations of *N,N´*-methylenebisacrylamide showing the amide (a) and aliphatic (b) regions with studied resonances indicated in blue.

Table S5. Chemical shifts and chemical shift changes of oxytocin proton resonances in the presence of increasing concentrations of *N,N´*-methylenebisacrylamide (BIS).

[BIS] (M)	**Chemical shift (ppm)** Leu NH	Gly NH	Asn NH	Ile NH	Asn NH_2 (a)	Gln NH_2 (a)	Gly NH_2 (a)	Gly NH_2 (b)	Asn NH_2 (b)	Asn3	Gln4	Pro3	Leu3
0	8,4873	8,4029	8,3291	7,8875	7,6177	7,5679	7,4161	7,0895	6,9324	2,8481	2,4192	2,3131	1,6146
0,00125	8,4873	8,4029	8,3292	7,8871	7,6177	7,5681	7,4161	7,0897	6,9325	2,8483	2,4193	2,3132	1,6151
0,00250	8,4874	8,4029	8,3294	7,8879	7,6180	7,5686	7,4164	7,0899	6,9328	2,8484	2,4194	2,3130	1,6151
0,00501	8,4870	8,4028	8,3290	7,8860	7,6179	7,5681	7,4161	7,0898	6,9326	2,8484	2,4193	2,3131	1,6152
0,01001	8,4862	8,4027	8,3286	7,8839	7,6183	7,5688	7,4168	7,0906	6,9326	2,8486	2,4194	2,3133	1,6151
0,02503	8,4864	8,4034	8,3294	7,8830	7,6196	7,5700	7,4180	7,0923	6,9339	2,8496	2,4200	2,3141	1,6157
0,03755	8,4856	8,4036	8,3286	7,8785	7,6204	7,5711	7,4189	7,0935	6,9344	2,8499	2,4200	2,3144	1,6165
0,05006	8,4849	8,4043	8,3283	7,8748	7,6213	7,5718	7,4197	7,0949	6,9347	2,8505	2,4202	2,3149	1,6169

[BIS] (M)	**Absolute chemical shift change (ppm)** Leu NH	Gly NH	Asn NH	Ile NH	Asn NH_2 (a)	Gln NH_2 (a)	Gly NH_2 (a)	Gly NH_2 (b)	Asn NH_2 (b)	Asn3	Gln4	Pro3	Leu3
0	0,0000	0,0000	0,0000	0,0000	0,0000	0,0000	0,0000	0,0000	0,0000	0,0000	0,0000	0,0000	0,0000
0,00125	0,0000	0,0000	0,0001	0,0004	0,0000	0,0002	0,0000	0,0002	0,0001	0,0002	0,0001	0,0001	0,0005
0,00250	0,0001	0,0000	0,0003	0,0004	0,0003	0,0007	0,0003	0,0004	0,0004	0,0003	0,0002	0,0001	0,0005
0,00501	0,0003	0,0001	0,0001	0,0015	0,0002	0,0002	0,0000	0,0003	0,0002	0,0003	0,0001	0,0000	0,0006
0,01001	0,0011	0,0002	0,0005	0,0036	0,0006	0,0009	0,0007	0,0011	0,0002	0,0005	0,0002	0,0002	0,0005
0,02503	0,0009	0,0005	0,0003	0,0045	0,0019	0,0021	0,0019	0,0028	0,0015	0,0015	0,0008	0,0010	0,0011
0,03755	0,0017	0,0007	0,0005	0,0090	0,0027	0,0032	0,0028	0,0040	0,0020	0,0018	0,0008	0,0013	0,0019
0,05006	0,0024	0,0014	0,0008	0,0127	0,0036	0,0039	0,0036	0,0054	0,0023	0,0024	0,0010	0,0018	0,0023

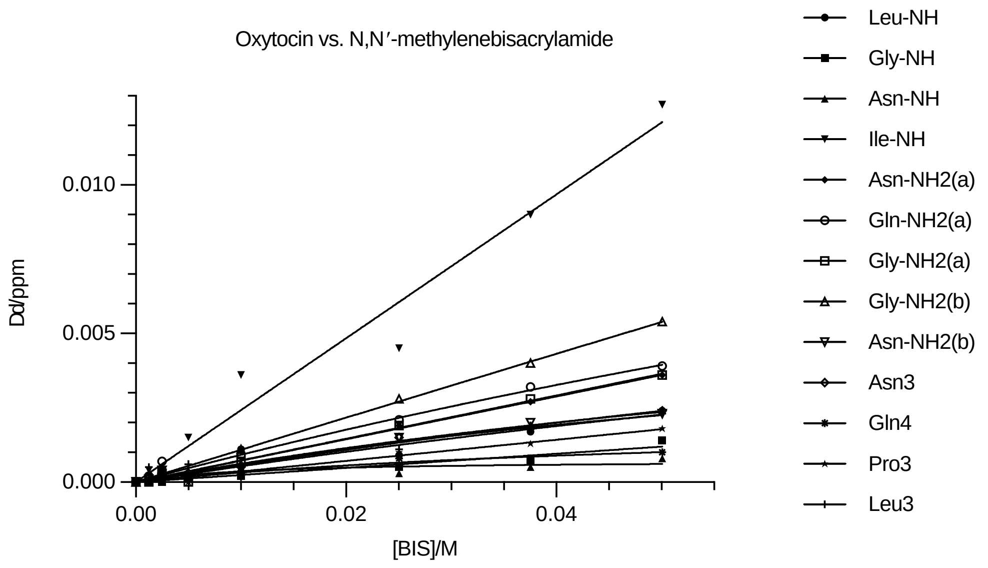

Figure S7. Plot of changes in chemical shift versus concentration of *N,N´*-methylenebisacrylamide (BIS). Curves correspond to the result of non-linear fitting.

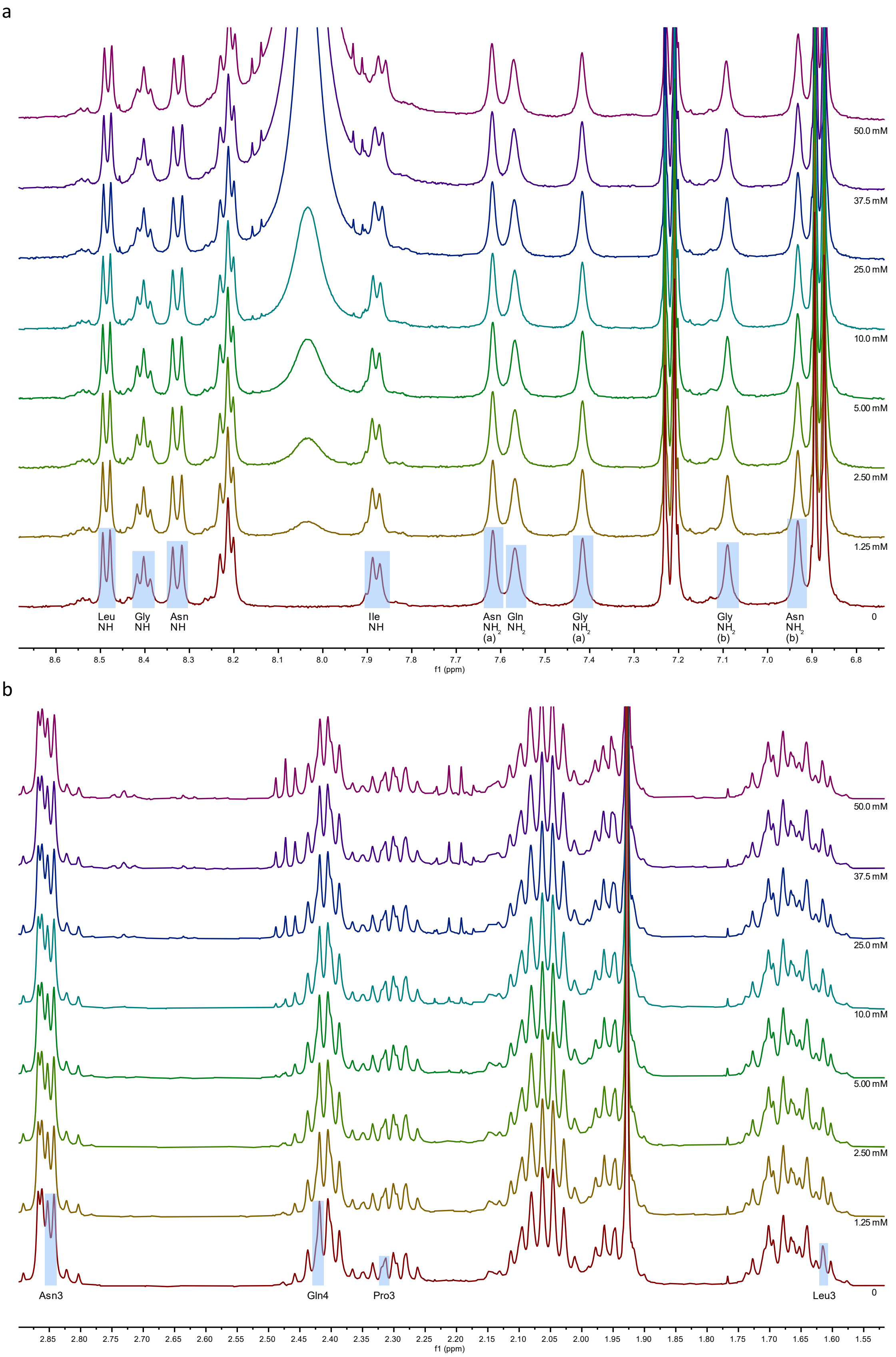

Figure S8. ^{1}H-NMR spectra of oxytocin in the presence of increasing concentrations of *N*-isopropylacrylamide showing the amide (a) and aliphatic (b) regions with studied resonances indicated in blue

Table S6. Chemical shifts and chemical shift changes of oxytocin proton resonances in the presence of increasing concentrations of *N*-isopropylacrylamide (NIPAM).

	**Chemical shift (ppm)**												
[NIPAM] M	Leu NH	Gly NH	Asn NH	Ile NH	Asn NH_2 (a)	Gln NH_2 (a)	Gly NH_2 (a)	Gly NH_2 (b)	Asn NH_2 (b)	Asn3	Gln4	Pro3	Leu3
0	8,4864	8,4026	8,3271	7,8796	7,6176	7,5680	7,4158	7,0901	6,9318	2,8476	2,4187	2,3130	1,6152
0,00125	8,4865	8,4027	8,3275	7,8804	7,6177	7,5681	7,4160	7,0901	6,9319	2,8474	2,4188	2,3130	1,6152
0,00250	8,4866	8,4028	8,3276	7,8810	7,6179	7,5682	7,4161	7,0901	6,9321	2,8478	2,4188	2,3129	1,6149
0,00501	8,4864	8,4026	8,3275	7,8809	7,6178	7,5682	7,4161	7,0902	6,9321	2,8477	2,4188	2,3130	1,6149
0,01001	8,4860	8,4025	8,3270	7,8787	7,6182	7,5685	7,4163	7,0905	6,9319	2,8476	2,4186	2,3130	1,6155
0,02503	8,4848	8,4020	8,3261	7,8751	7,6186	7,5695	7,4165	7,0914	6,9317	2,8481	2,4184	2,3130	1,6158
0,03755	8,4842	8,4026	8,3262	7,8743	7,6192	7,5700	7,4170	7,0921	6,9318	2,8477	2,4185	2,3135	1,6159
0,05006	8,4828	8,4026	8,3244	7,8669	7,6195	7,5708	7,4173	7,0929	6,9313	2,8474	2,4179	2,3134	1,6161

	**Absolute chemical shift change (ppm)**												
[NIPAM] M	Leu NH	Gly NH	Asn NH	Ile NH	Asn NH_2 (a)	Gln NH_2 (a)	Gly NH_2 (a)	Gly NH_2 (b)	Asn NH_2 (b)	Asn3	Gln4	Pro3	Leu3
0	0,0000	0,0000	0,0000	0,0000	0,0000	0,0000	0,0000	0,0000	0,0000	0,0000	0,0000	0,0000	0,0000
0,00125	0,0001	0,0001	0,0004	0,0008	0,0001	0,0001	0,0002	0,0000	0,0001	0,0002	0,0001	0,0000	0,0000
0,00250	0,0002	0,0002	0,0005	0,0014	0,0003	0,0002	0,0003	0,0000	0,0003	0,0002	0,0001	0,0001	0,0003
0,00501	0,0000	0,0000	0,0004	0,0013	0,0002	0,0002	0,0003	0,0001	0,0003	0,0001	0,0001	0,0000	0,0003
0,01001	0,0004	0,0001	0,0001	0,0009	0,0006	0,0005	0,0005	0,0004	0,0001	0,0000	0,0001	0,0000	0,0003
0,02503	0,0016	0,0006	0,0010	0,0045	0,0010	0,0015	0,0007	0,0013	0,0001	0,0005	0,0003	0,0000	0,0006
0,03755	0,0022	0,0000	0,0009	0,0053	0,0016	0,0020	0,0012	0,0020	0,0000	0,0001	0,0002	0,0005	0,0007
0,05006	0,0036	0,0000	0,0027	0,0127	0,0019	0,0028	0,0015	0,0028	0,0005	0,0002	0,0008	0,0004	0,0009

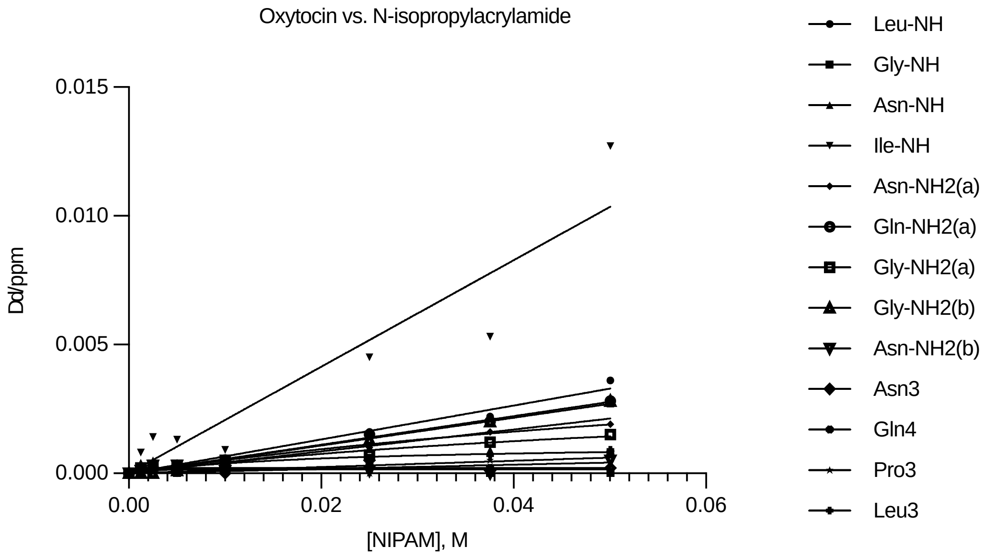

Figure S9. Plot of changes in chemical shift versus concentration of *N*-isopropylacrylamide (NIPAM). Curves correspond to the result of non-linear fitting.

a

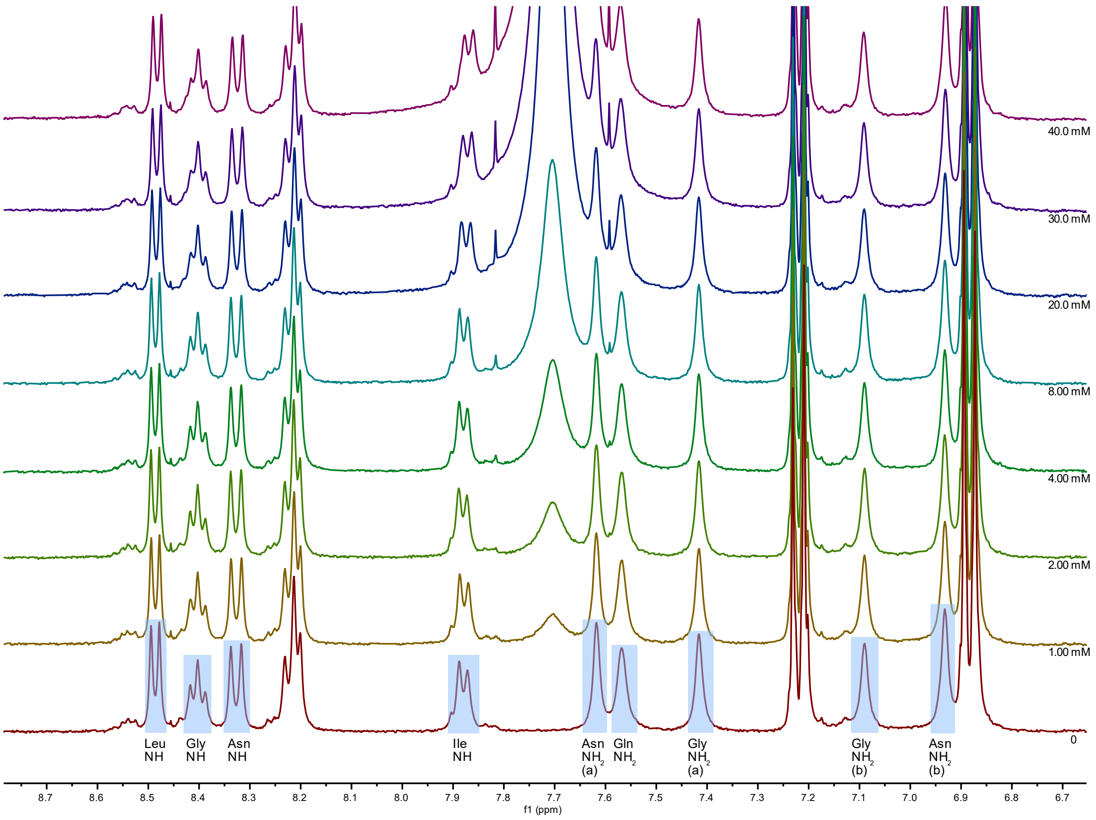

b

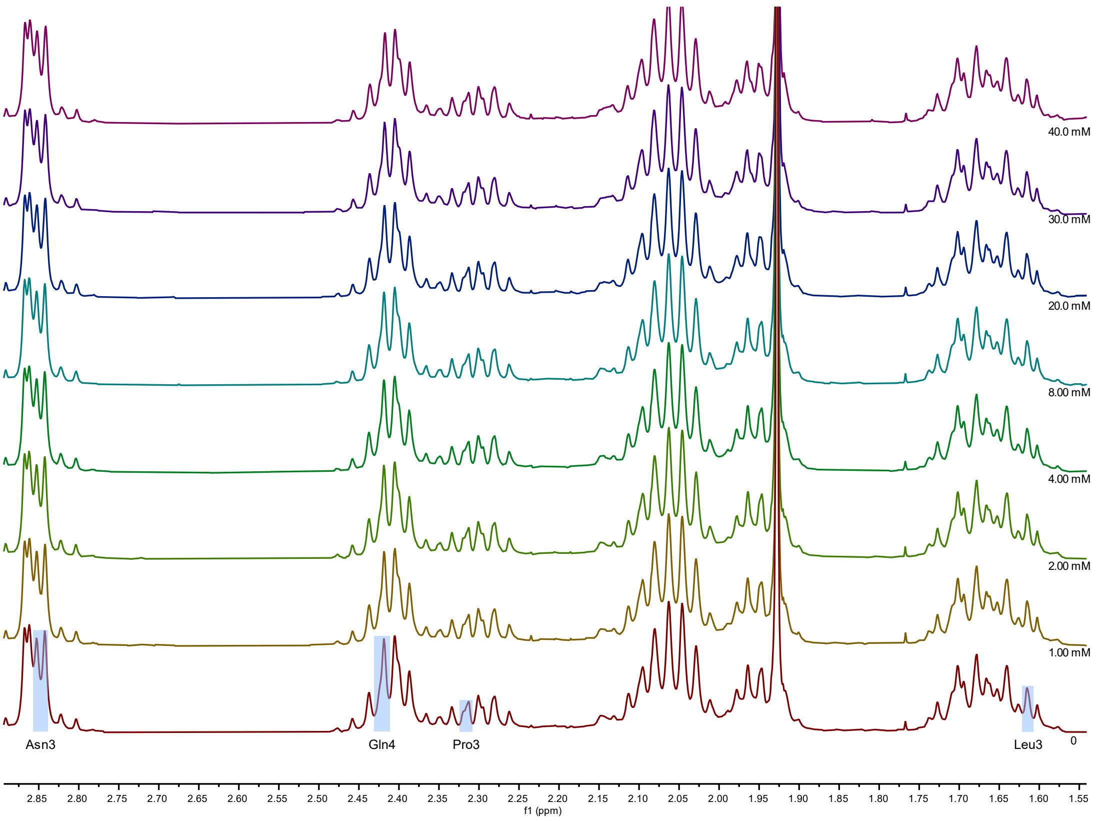

Figure S10. ^{1}H-NMR spectra of oxytocin in the presence of increasing concentrations of *N*-*t*-butylacrylamide showing the amide (a) and aliphatic (b) regions with studied resonances indicated in blue

Table S7. Chemical shifts and chemical shift changes of oxytocin proton resonances in the presence of increasing concentrations of *N*-*t*-butylacrylamide (TBAM).

[TBAM][a] M	Chemical shift (ppm)												
	Leu NH	Gly NH	Asn NH	Ile NH	Asn NH_2 (a)	Gln NH_2 (a)	Gly NH_2 (a)	Gly NH_2 (b)	Asn NH_2 (b)	Asn3	Gln4	Pro3	Leu3
0	8,4864	8,4026	8,3271	7,8796	7,6176	7,5680	7,4158	7,0901	6,9318	2,8476	2,4187	2,3130	1,6152
0,00100	8,4861	8,4024	8,3267	7,8784	7,6178	7,5682	7,4160	7,0902	6,9317	2,8474	2,4185	2,3127	1,6153
0,00200	8,4865	8,4026	8,3274	7,8806	7,6178	7,5680	7,4159	7,0900	6,9319	2,8477	2,4187	2,3128	1,6153
0,00399	8,4862	8,4025	8,3271	7,8798	7,6178	7,5681	7,4160	7,0901	6,9318	2,8475	2,4187	2,3130	1,6150
0,00799	8,4858	8,4023	8,3271	7,8791	7,6180	7,5686	7,4161	7,0903	6,9317	2,8476	2,4186	2,3128	1,6149
0,01997	8,4844	8,4017	8,3257	7,8745	7,6183	7,5693	7,4163	7,0908	6,9312	2,8472	2,4181	2,3130	1,6156
0,02996	8,4834	8,4018	8,3251	7,8718	7,6188	7,5698	7,4162	7,0911	6,9308	2,8470	2,4178	2,3130	1,6156
0,03994	8,4824	8,4014	8,3243	7,8690	7,6193	7,5707	7,4165	7,0915	6,9305	2,8468	2,4174	2,3129	1,6153

[TBAM] [a] M	Absolute chemical shift change (ppm)												
	Leu NH	Gly NH	Asn NH	Ile NH	Asn NH_2 (a)	Gln NH_2 (a)	Gly NH_2 (a)	Gly NH_2 (b)	Asn NH_2 (b)	Asn3	Gln4	Pro3	Leu3
0	0,0000	0,0000	0,0000	0,0000	0,0000	0,0000	0,0000	0,0000	0,0000	0,0000	0,0000	0,0000	0,0000
0,00100	0,0003	0,0002	0,0004	0,0012	0,0002	0,0002	0,0002	0,0001	0,0001	0,0002	0,0002	0,0003	0,0001
0,00200	0,0001	0,0000	0,0003	0,0010	0,0002	0,0000	0,0001	0,0001	0,0001	0,0001	0,0000	0,0002	0,0001
0,00399	0,0002	0,0001	0,0000	0,0002	0,0002	0,0001	0,0002	0,0000	0,0000	0,0001	0,0000	0,0000	0,0002
0,00799	0,0006	0,0003	0,0000	0,0005	0,0004	0,0006	0,0003	0,0002	0,0001	0,0000	0,0001	0,0002	0,0003
0,01997	0,0020	0,0009	0,0014	0,0051	0,0007	0,0013	0,0005	0,0007	0,0006	0,0004	0,0006	0,0000	0,0004
0,02996	0,0030	0,0008	0,0020	0,0078	0,0012	0,0018	0,0004	0,0010	0,0010	0,0006	0,0009	0,0000	0,0004
0,03994	0,0040	0,0012	0,0028	0,0106	0,0017	0,0027	0,0007	0,0014	0,0013	0,0008	0,0013	0,0001	0,0001

[a] max concentration was 40 mM due to solubility

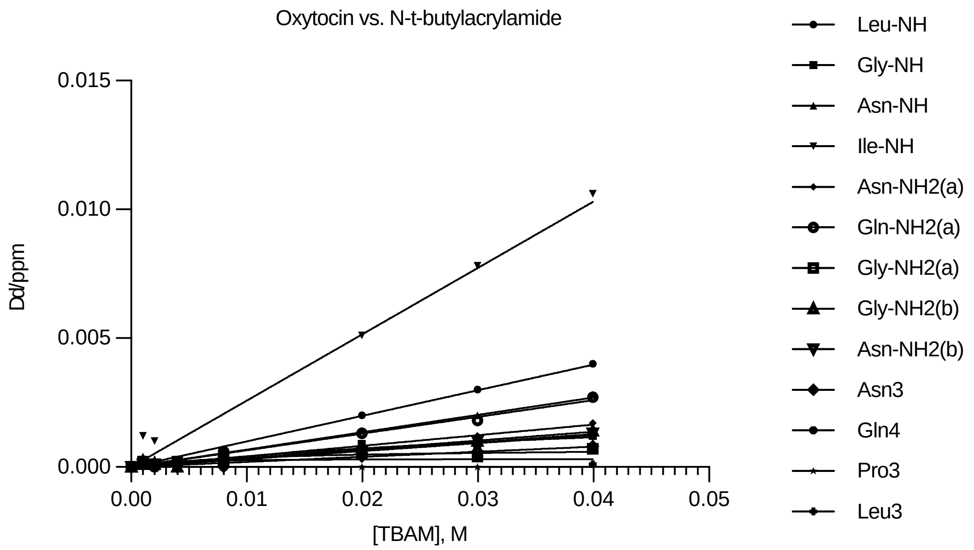

Figure S11. Plot of changes in chemical shift versus concentration of *N*-*t*-butylacrylamide (TBAM). Curves correspond to the result of non-linear fitting.

Molecular dynamics simulations

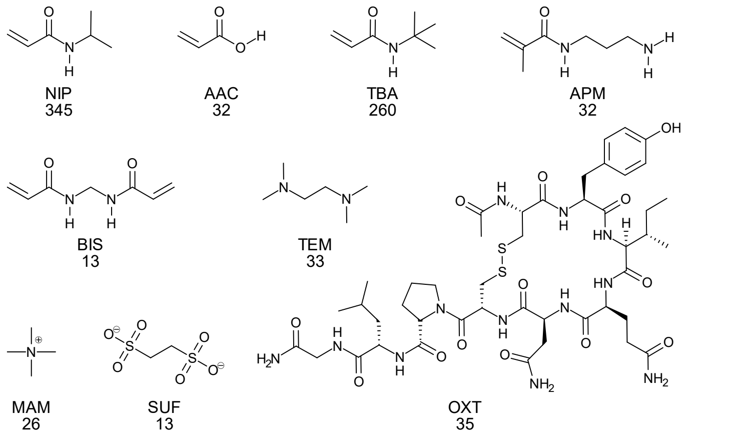

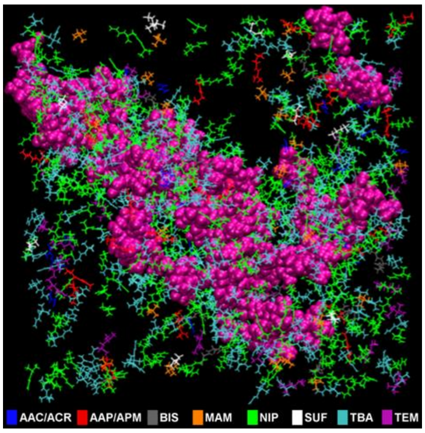

Figure S12. Left: Number of each molecule used in the molecular dynamics simulations and the abbreviations used in the analysis modules: NIP – *N*-isopropylacrylamide, AAC – acrylic acid, TBA – *N-tert*-butylacrylamide, APM – *N*-3-aminopropyl methacrylamide, BIS – *N,N'*-methylenebisacrylamide, TEM – *N,N,N',N'*-tetramethylenediamine, MAM – tetramethylammonium, SUF – ethane-1,2-disulfonate and OXT – oxytocin. MAM and SUF were included to mimic the initiator ammonium persulfate which did not parametrize well in the protein.ff14SB force field. In addition, 30 000 water molecules were included. Right: Snapshot after 100 ns production phase simulation. Oxytocin is visualized as pink van der Waals representations, remaining components using stick representations. Water molecules have been omitted for clarity.

Hydrogen bond analysis

Results obtained using the HBOND routine of CPPTRAJ describe the fraction of total simulation time where pairs of atoms analyzed fulfil the default distance and angle criteria defined as a hydrogen bond. Obtained fractions for each pair of atoms were summarized, converted to percent of total simulation time (occupancy) and averaged against the number of molecules, producing an average occupancy for the interaction. These results were summarized by adding together all observed averaged occupancies between all identified hydrogen bond donor/acceptor pairs of two molecules, Table S8. Interactions with water molecules are excluded as these analyses were nonconclusive (all analyses were not possible to execute due to hardware restrictions).

Table S8. Average hydrogen bond occupancy between molecule pairs

	OXT	AAC	APM	NIP	TBA	BIS	TEM	SUF
**OXT**	33,55	3,83	5,24	68,30	44,28	4,09	0,21	0,66
**AAC**	3,83	0,06	0,39	5,07	4,00	0,25	0,11	0,38
**APM**	5,24	0,39	0,11	2,33	1,28	0,34	0,01	0,07
**NIP**	68,30	5,07	2,33	1,81	2,18	6,46	0,00	0,03
**TBA**	44,28	4,00	1,28	2,18	0,71	3,91	0,00	0,02
**BIS**	4,09	0,25	0,34	6,46	3,91	0,17	0,01	0,59
**TEM**	0,21	0,11	0,01	0,00	0,00	0,01	-	-
**SUF**	0,66	0,38	0,07	0,03	0,02	0,59	-	-

Occupancy values reflects interactions between molecules but does not differentiate between frequent short interactions or fewer but more stable interactions. Lifetime data obtained from hydrogen bond analyses results are hence valuable additions to the degree of interaction presented (Table S9).

Interactions when AAC acts as H donor and SUF or TEM as acceptors displays the most stable interactions. These interactions are highly infrequent (Table S8) though if formed predicted to be stable. This highlights the complicated nature of interpretation of results in these systems. Importantly, interactions between the acidic proton of AAC and any acceptor constitute the most stable interactions

Table S9. Average lifetimes of hydrogen bond interactions.[a]

		H-donor					
		AAC	APM	BIS	NIP	OXT	TBA
H-acceptor	AAC	2,22	2,46	2,45	2,24	2,92	2,39
	APM	2,46	1,12	1,22	1,17	1,34	1,14
	BIS	2,45	1,22	1,39	1,26	1,58	1,33
	NIP	2,24	1,17	1,26	1,22	1,49	1,19
	OXT	2,92	1,34	1,58	1,49	1,74	1,52
	SUF	6,73	1,27	1,66	1,46	2,02	1,43
	TBA	2,39	1,14	1,33	1,19	1,52	1,17
	TEM	7,05	1,17	1,35	1,08	1,75	1,00

[a] The observed lifetimes for analyzed pairs of atoms forming a hydrogen bond in picoseconds (ps), initially averaged against the number of observations (number of registered hydrogen bond events) for each analyzed pair of interacting atoms. The lifetimes, average per interaction between two interacting atoms, were summarized for all interactions between two molecules and then averaged against the number of analyzed donor and acceptor atoms on both interacting molecules. The values are average lifetimes for any average registered hydrogen bond between any donor/acceptor on the interacting molecules.

Radial distribution function and grid density analyses were performed to add additional insights into potential arrangements of associating molecules, providing details regarding potential non-hydrogen bond interactions or areas of preferred association. Results from these analyses are presented in Fig. S13.

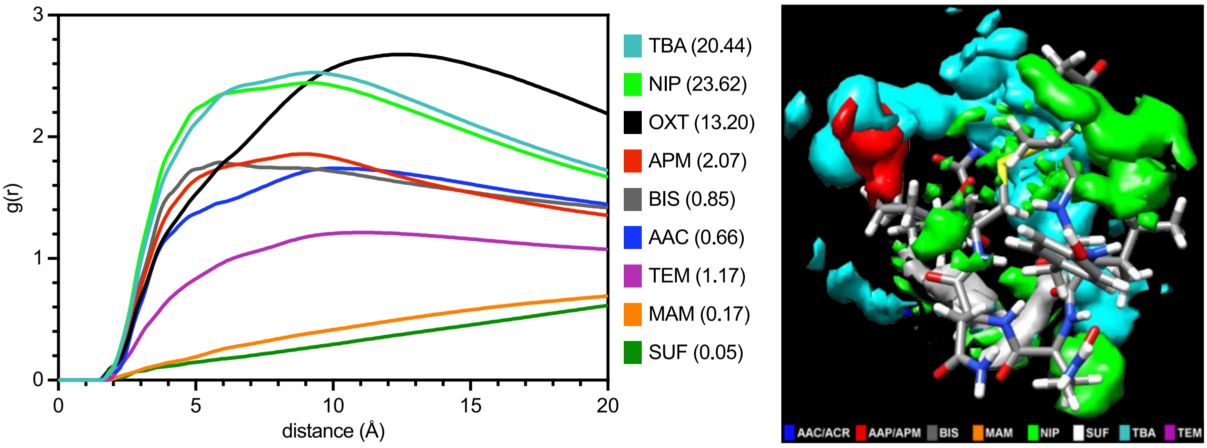

Figure S13. Left: Radial distribution functions for polymer mixture components around oxytocin. Numbers in parentheses are the integrated RDF values, corresponding to the relative local numeric density of each molecule within a 7.55 Å cutoff radius. Right: Grid density map plot around oxytocin. Only one oxytocin structure displayed for clarity.

Dynamic light scattering

Table S10. Physical dimensions and yields of different batches of O-NPs using DLS

Batch no.	Z-average (nm)	PDI	Yield (mg)
**1**	275 ± 13	0.304 ± 0.022	1.95
**2**	215 ± 11	0.223 ± 0.006	1.55
**3**	253 ± 39	0.301± 0.029	1.90

X-ray photoelectron spectroscopy

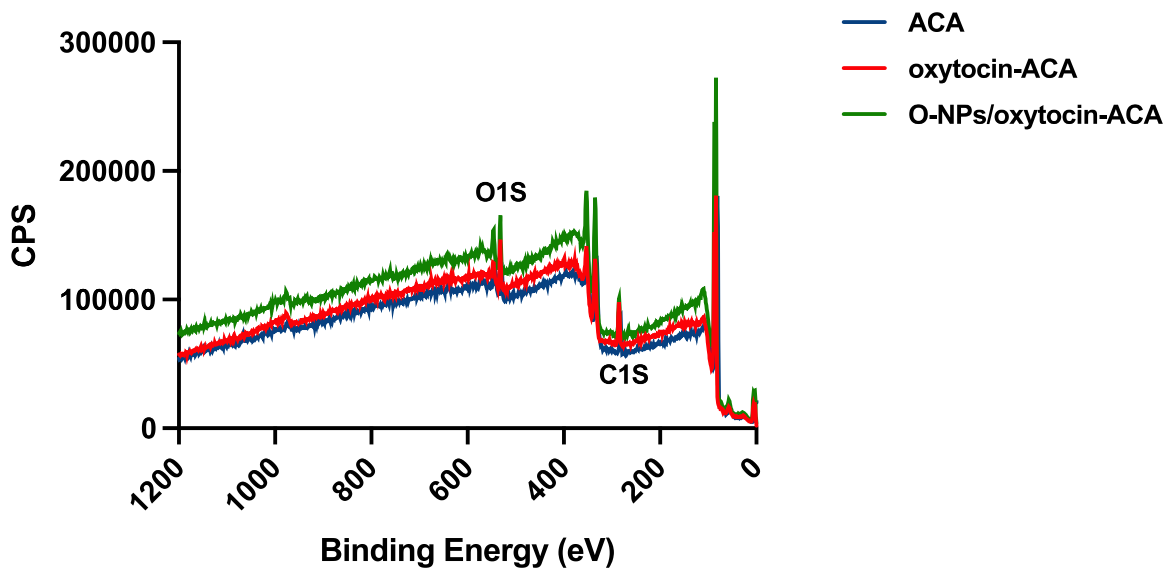

Figure S14. XPS survey spectrum of ACA modified LNB chips, oxytocin immobilized on ACA modified LNB chips and after binding of O-NPs

Table S11. Area and intensity obtained by high resolution C1S XPS spectra

Samples	Area	Intensity
**O-NPs/oxytocin-ACA**	59788	21398
**oxytocin-ACA**	54226	15933
**ACA**	50361	15724

Reflection adsorption infrared spectroscopy

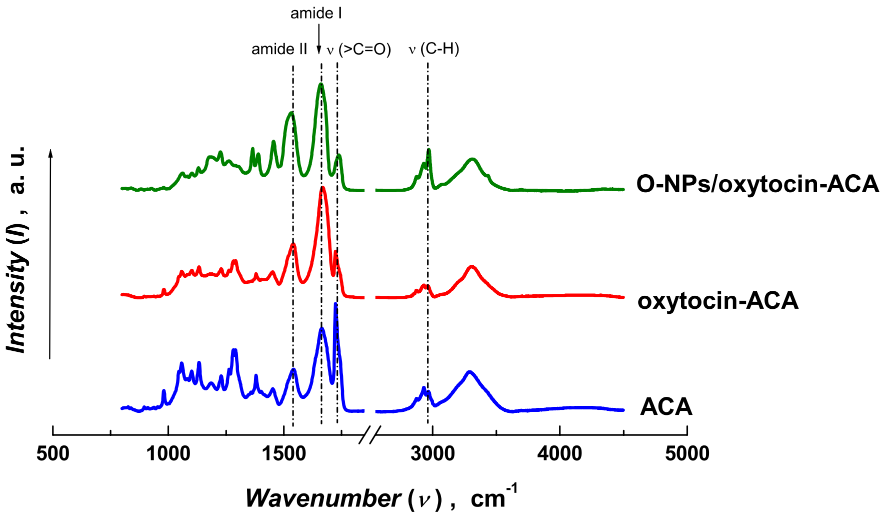

Figure S15. RAIRS spectra recorded in the course of O-NPs binding strategy (ACA, oxytocin-ACA and O-NPs oxytocin-ACA), prepared on functionalized gold surfaces

Quartz crystal microbalance studies

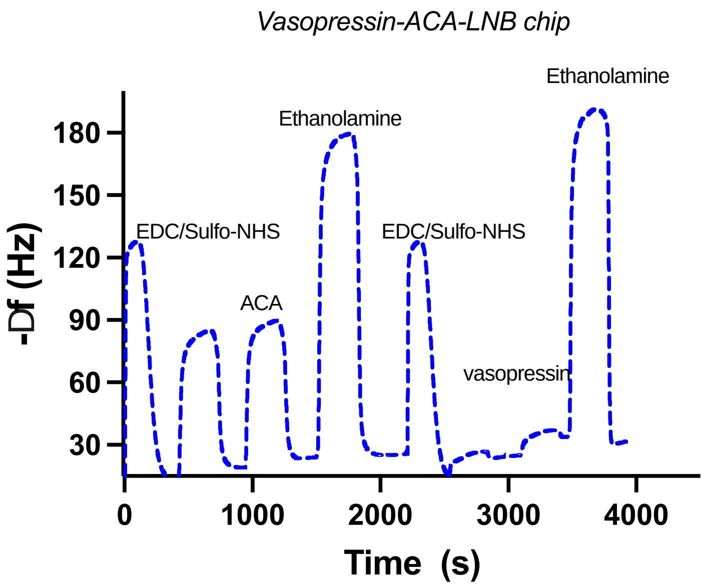

Figure S16. QCM trace for the immobilization of vasopressin.

Determination of binding constants

The affinity complexes of O-NPs with oxytocin (O-NPs|Oxy) can be expressed as an equilibrium by equation S2, described by association (k_a) and dissociation rate constants (k_d).

$$\text{O-Nps} + \text{Oxytocin} \underset{k_d}{\overset{k_a}{\rightleftharpoons}} \text{O-NPs}|\text{Oxy} \quad \text{(Eq. S2)}$$

The rate of formation of the O-NPs|Oxy complex can be related to the resonant frequency change (f) and injected O-NPs concentration, c.

$$d[\text{O-NPs}|\text{Oxy}]/dt = -df/dt = k_a (f_{\max} - f)\, c - k_d f \quad \text{(Eq. S3)}$$

where f_{max} is the maximum change in the resonant frequency of the oxytocin immobilized LNB chips due to injection of O-NPs with concentration c. Upon integrating with time t and substituting $k_a c f_{max}/(k_a c + k_d)$ and $k_{obs} = k_a c + k_d$, equation S3 can be simplified to equation S4:

$$f_{eq} = f_{\max} [1 - \exp(-k_{obs}\, t)] \quad \text{(Eq. S4)}$$

The initial part of resonant frequency change with time, recorded immediately after FIA injection of the O-NPs, has been fitted to equation S4. The calculated k_{obs} was plotted against the concentration of O-NPs. From the plot, the k_d value was determined using one site specific binding analysis using PRISM software.
